# Abrogation of Oncogenic RAS Signaling by a RAS(ON) Inhibitor Doublet Primes Immune-Refractory *KRAS*^*G12C*^-Mutant NSCLC for Immune Checkpoint Blockade

**DOI:** 10.1158/2159-8290.CD-25-1616

**Published:** 2026-02-11

**Authors:** Xing Wei, Cristina Blaj, M. Ali Al-Radhawi, Lick Pui Lai, Benjamin J. Maldonato, Yu Chi Yang, Lillian Seu, Harika Gundlapalli, Lingyan Jiang, Mariela A. Moreno Ayala, Jessica N. Spradlin, Brett Garrick, Shurui Cai, Avery Salmon, Anna Pham, Sean Bredeson, Rich Liang, Ciara Helland, James W. Evans, Mark P. Labrecque, Xinxing Yu, Avian Hyun Joo Song, Nuntana Dinglasan, Linh Tran, Alice Kumamoto, Lilit Grigoryan, Angeliki Malliri, Katherine D. Brown, Mathew Carter, Kathryn L. Simpson, Philip A. Crosbie, Melanie Galvin, Stephanie Chang, Yue Huang, Nataliya Tovbis Shifrin, Ximo Pechuan-Jorge, Rashi Raghulan, Yongxian Zhuang, Darryl I. Coles, Caroline Dive, Ida Aronchik, Matthew Holderfield, Colin R. Lindsay, Zhengping Wang, Zhican Wang, Mallika Singh, Jacqueline A.M. Smith, Jingjing Jiang, Elsa Quintana

**Affiliations:** 1 Revolution Medicines, Inc., Redwood City, California.; 2 Division of Cancer Sciences, University of Manchester, Manchester, United Kingdom.; 3 Cancer Research UK Lung Cancer Centre of Excellence, Manchester, United Kingdom.; 4 The Christie NHS Foundation Trust, Manchester, United Kingdom.; 5 SCLC Biology Group, Cancer Research UK Manchester Institute, University of Manchester, Manchester, United Kingdom.; 6 Cancer Research UK National Biomarker Centre, University of Manchester, Manchester, United Kingdom.; 7 Division of Immunology, Immunity to Infection and Respiratory Medicine, University of Manchester, Manchester, United Kingdom.; 8 North West Lung Centre, Manchester University NHS Foundation Trust, Manchester, United Kingdom.

## Abstract

To address RAS pathway hyperactivation and targeted therapy resistance in *KRAS*^*G12C*^-mutant non–small cell lung cancer (NSCLC), we evaluated the potential of the RAS(ON) G12C-selective covalent inhibitor elironrasib and the RAS(ON) multi-selective inhibitor daraxonrasib combination to maximize RAS pathway suppression and forestall pathway reactivation in a series of preclinical models. We demonstrate that the RAS(ON) inhibitor doublet induces profound and sustained tumor regressions and overcomes the increased RAS pathway oncogenic flux that underlies resistance to inactive state–selective KRAS^G12C^ inhibitors in NSCLC. Additionally, in immune-competent preclinical models, the RAS(ON) inhibitor doublet enhances tumor immune recognition by boosting antigen presentation and remodeling the suppressive tumor microenvironment, thus promoting immune-dependent complete regressions and sensitization of an immunorefractory model to checkpoint blockade. Collectively, these findings provide a preclinical rationale for the evaluation of a targeted RAS(ON) inhibitor doublet therapy regimen in combination with immune checkpoint blockade (ICB) in patients with *KRAS*^*G12C*^-mutant NSCLC.

**Significance:**

The combination of a RAS(ON) G12C-selective and RAS(ON) multi-selective inhibitor mitigates clinical resistance mechanisms to KRAS^G12C^(OFF) inhibitors and enhances tumor immune recognition, overcoming ICB resistance. These preclinical findings highlight the potential for a RAS(ON) targeted therapy regimen in combination with anti–PD-(L)1 in patients with *KRAS*^*G12C*^-mutant NSCLC.

*See related commentary by Molina-Arcas and Downward, p. 1044*

## Teaser

A RAS(ON) inhibitor doublet overcomes resistance to KRAS^G12C^-targeted therapy and enhances tumor immunogenicity, sensitizing immune-refractory KRAS^G12C^-mutant non-small cell lung cancer to immunotherapy.

## Introduction

Oncogenic mutations in the canonical RAS genes, *KRAS*, *NRAS*, and *HRAS*, drive malignant growth in various human cancers, with the highest prevalence in pancreatic ductal adenocarcinoma (PDAC, >90%), colorectal cancer (∼50%), and non–small cell lung cancer (NSCLC, ∼30%), accounting for approximately 200,000 new cancers diagnosed in the United States each year (1, 2). As one of the common hotspot mutations in *KRAS*, the glycine-to-cysteine amino acid substitution at residue 12 (G12C) is predominant in patients with NSCLC harboring *KRAS* mutations, accounting for about 40% of these cases (1).

The discovery and FDA approval of the KRAS^G12C^ mutant-selective inhibitors sotorasib (Lumakras) and adagrasib (Krazati) have reshaped the therapeutic landscape of *KRAS*^*G12C*^-mutant advanced NSCLC (3–7). These inhibitors selectively target the KRAS^G12C^ protein in the GDP-bound inactive state [KRAS^G12C^(OFF)] via covalent modification of the mutated cysteine residue. However, the clinical outcomes from KRAS^G12C^(OFF) inhibitor treatment are limited by primary and acquired resistance mechanisms, indicating the need for improved therapeutic agents and effective combinations (8, 9). To address the high unmet medical need in RAS-addicted cancers, we have developed a series of tri-complex inhibitors selective for the GTP-bound or active state of RAS [RAS(ON)]. This includes the investigational agents elironrasib, a RAS(ON) G12C-selective covalent inhibitor, and daraxonrasib, a RAS(ON) multi-selective inhibitor (10–13). Both elironrasib and daraxonrasib have demonstrated profound antitumor activity as monotherapies in preclinical models and, more recently, evidence of preliminary clinical activity in patients with advanced solid tumors harboring RAS mutations at tolerated dose levels (10–15).

By directly targeting RAS^G12C^(ON), elironrasib is anticipated to mitigate some of the clinical resistance mechanisms that have been observed with KRAS^G12C^(OFF) inhibitors (10). In preclinical models, RAS(ON) G12C-selective inhibitors are less susceptible to RAS pathway hyperactivation resulting from increased levels of KRAS^G12C^(ON) protein, upstream RTK activation, and/or switch II binding pocket mutations in KRAS^G12C^, all of which can confer resistance to the KRAS^G12C^(OFF) inhibitors (9, 11). Consistent with these observations, preliminary clinical evidence for the activity of elironrasib in patients who have progressed on a KRAS^G12C^(OFF) inhibitor has been reported (15). However, as a RAS(ON) G12C-selective inhibitor, elironrasib cannot address other potential escape mechanisms such as wild-type (WT) RAS-mediated pathway reactivation via H/N or KRAS, RTK alterations, or emergent secondary *RAS* mutations. These should be susceptible to inhibition by daraxonrasib which exhibits broad spectrum inhibition of both mutant and WT K, N, and HRAS variants (12, 13, 16). Therefore, a RAS(ON) inhibitor doublet combination of elironrasib with daraxonrasib may offer improved therapeutic outcomes for patients with *KRAS*^*G12C*^-mutant NSCLC when compared with KRAS^G12C^-selective inhibitor monotherapy. This combination should maximize tumor-intrinsic RAS pathway suppression and be less vulnerable to the diversity of acquired clinical resistance mechanisms that have been observed in patients who have progressed on a KRAS^G12C^(OFF) inhibitor (9, 17, 18).

First-line therapy for patients with advanced NSCLC, including those harboring *KRAS* mutations, includes immune checkpoint blockade (ICB) with anti–PD-1/anti–PD-L1 immunotherapy, often in combination with chemotherapy and sometimes with anti–CTLA-4 agents (19–22). Patients with *KRAS*^*G12C*^-mutant NSCLC, which is commonly linked to smoking (23), tend to exhibit higher tumor mutational burden (TMB) and elevated PD-L1 expression compared with non-G12C *KRAS* mutants, possibly contributing to increased sensitivity to immunotherapies (24). However, a significant subset of these patients does not benefit from ICB, with MHC class I downregulation being a common mechanism of immune evasion in NSCLC (25–28). In addition, mutant KRAS has been shown to sustain an immunosuppressive tumor microenvironment (TME) and facilitate cancer cell immune escape by several mechanisms, including secretion of anti-inflammatory chemokines and cytokines, differentiation and infiltration of immunosuppressive regulatory cells, inhibition of T-cell activation, and suppression of cytotoxic CD8^+^ T cell–mediated tumor killing via MHC class I downregulation and PD-L1 upregulation on the tumor cell membrane (29). Preclinical studies have shown that KRAS-targeted therapies can partially reverse this immunosuppressive TME by enhancing adaptive immune responses (30–32), prompting multiple clinical trials to evaluate the combination of KRAS^G12C^(OFF) mutant-selective inhibitors with ICB in *KRAS*^*G12C*^-mutant NSCLC (33, 34). Early signs of clinical benefit have been reported for some of these combinations despite the apparent challenges encountered identifying tolerable combination regimens (33, 34). Preclinical studies have suggested that the potential benefits of an ICB plus a mutant-selective RAS inhibitor combination may be limited to immunogenic NSCLC tumors, characterized by high lymphocyte infiltration, and not extend to immune-excluded lung tumors (30). Therefore, more effective combination therapies that can increase T-cell infiltration and enhance tumor recognition are needed to broaden the activity of ICB in NSCLC.

In this study, we investigated the therapeutic potential of the RAS(ON) inhibitor doublet of elironrasib plus daraxonrasib in preclinical models of *KRAS*^*G12C*^-mutant NSCLC that are refractory to KRAS^G12C^ inhibitor monotherapy, including those harboring genomic alterations known to confer clinical resistance to KRAS^G12C^(OFF) inhibitors. We demonstrate that the RAS(ON) inhibitor doublet can overcome these barriers, driving sustained RAS pathway suppression and deeper and more durable responses than either agent alone. These effects were further supported by pharmacokinetics (PK)/target engagement (TE)/pharmacodynamics (PD) modeling of oncogenic KRAS^G12C^ inhibition. In addition, we show that maximizing RAS pathway suppression with a RAS(ON) inhibitor doublet sensitizes ICB-refractory NSCLC tumors to anti–PD-1 by promoting tumor immune recognition and activating adaptive immunity, driving immune-dependent long-term complete tumor responses. Together, these preclinical data support the clinical evaluation of a triplet combination strategy with elironrasib, daraxonrasib, and ICB as a targeted therapy regimen for patients with *KRAS*^*G12C*^-mutant NSCLC.

## Results

### The RAS(ON) Inhibitor Doublet Induces Deep and Durable Responses in Refractory *KRAS*^*G12C*^-Mutant NSCLC Models

**Figure 1.**
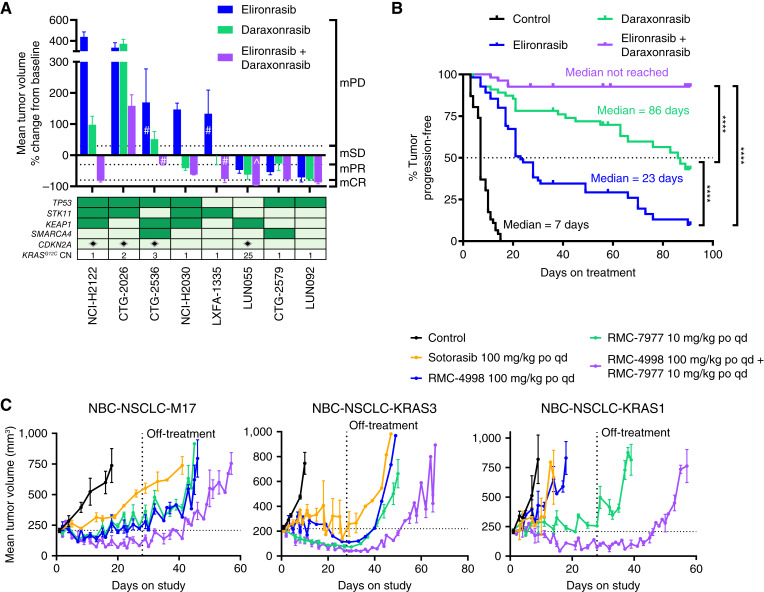
RAS(ON) inhibitor doublet induces deep and durable responses in *KRAS*^*G12C*^-mutant NSCLC xenograft models. **A,** Tumor response waterfall plot of eight *KRAS*^*G12C*^-mutant NSCLC subcutaneous xenograft models upon daily treatment of elironrasib at 30, 100, or 200 mg/kg and daraxonrasib at 25 mg/kg as single agents or in combination (*n* = 3–15 per group in each model). Average % mean tumor volume change ± SEM from baseline at response calling date are shown. mRECIST criteria were used to call tumor response as indicated on the right-hand side of the waterfall plot. Oncoplots illustrating gene alterations and expression levels in critical genes linked to the clinicopathologic characteristics of the indicated models are shown below the waterfall. Color coding represents dark green for mutations and light green for the absence of mutations. The symbol denotes that mRNA of corresponding genes is not expressed, defined as having a gene expression value of ≤0.5 CPM. The bottom row indicates the *KRAS*^*G12C*^ gene CN in each model as assessed by digital PCR. ^ and # symbols indicate that elironrasib was dosed at 30 or 200 mg/kg in specific group, respectively. **B,** Kaplan–Meier analysis of time to tumor doubling on treatment in individual tumor-bearing animals from eight *KRAS*^*G12C*^-mutant NSCLC subcutaneous xenograft models upon daily treatment of vehicle, elironrasib at 30, 100, or 200 mg/kg and daraxonrasib at 25 mg/kg as single agents or in combination for up to 90 days (*n* = 46 animals in control, *n* = 55 in elironrasib and daraxonrasib single agent groups, *n* = 54 in elironrasib and daraxonrasib doublet combination group). Time to event was determined by the time on treatment until tumor volume doubling from baseline on the survival plot by Kaplan–Meier analysis. Log-rank test was used to compare specific treatment groups (****, *P* < 0.0001). **C,** Antitumor activity of sotorasib, RMC-4998, RMC-7977, and the combination of RMC-4998 with RMC-7977 in three *KRAS*^*G12C*^-mutant NSCLC subcutaneous PDX models, including NBC-NSCLC-M17 (*n* = 5–7 per group), NBC-NSCLC-KRAS3 (*n* = 5–6 per group), and NBC-NSCLC-KRAS1 (*n* = 4–7 per group). Tumor-bearing mice were treated with vehicle or inhibitors (sotorasib at 100 mg/kg orally every day, RMC-4998 at 100 mg/kg orally every day, RMC-7977 at 10 mg/kg orally every day, and the combination of RMC-4998 at 100 mg/kg orally every day plus RMC-7977 at 10 mg/kg orally every day) for up to 28 days followed by off-treatment measurements. Mean tumor volumes of each group were plotted over the course of treatment. The horizontal dotted line indicates the initial average tumor volume. The vertical dotted line indicates treatment stop. Error bars, SEM. CN, copy number; po, orally; qd, every day.

We previously reported that elironrasib alone demonstrated profound antitumor activity *in vivo* with tumor regressions across a large panel of *KRAS*^*G12C*^-mutant NSCLC cell line–derived xenograft (CDX) and patient-derived xenograft (PDX) models, with overall response rate (ORR) = 72% (18/25) as assessed by a modified Response Evaluation Criteria in Solid Tumors for xenografts (mRECIST; see “Methods” for details; refs. 11, 35). Here, we report that these responses were relatively durable in a long-term treatment experiment (up to 90 days) as shown in a Kaplan–Meier analysis, in which tumor progression was defined as individual tumor volume doubling from baseline (35) and the median progression-free survival (mPFS) for elironrasib was not reached (Supplementary Fig. S1A; Supplementary Table S1). For the present study, we focused on a cohort (*n* = 8) of models from this panel that exhibited suboptimal responses to elironrasib monotherapy [ORR = 38% (3/8); mPFS = 23 days]. This subset harbors comutations, e.g., *TP53*, *STK11*, *KEAP1*, *SMARCA4*, and *CDKN2A* loss, that are not only common in *KRAS*^*G12C*^-mutant NSCLC (Fig. 1A) but, in some cases, have been implicated in driving reduced and/or refractory responses to KRAS^G12C^(OFF) inhibition (36, 37). Of note, one of these models (LUN055) also exhibited an increase in *KRAS*^*G12C*^ copy number at baseline; amplification of mutant *KRAS*^*G12C*^ has been reported as a driver of acquired resistance to inactive state inhibition (Fig. 1A; Supplementary Fig. S1B; ref. 38). Daraxonrasib monotherapy treatment resulted in a similar ORR = 38% (3/8) as compared with elironrasib in this cohort but more durable antitumor activity with a significantly improved mPFS of 86 days (Fig. 1A and B; Supplementary Tables S1 and S2). Notably, the RAS(ON) inhibitor doublet regimen significantly improved antitumor activity as compared with either monotherapy, with ORR = 75% (6/8) (Fig. 1A; Supplementary Table S2) and mPFS not reached during a treatment interval of up to 90 days (Fig. 1B; Supplementary Fig. S1C; Supplementary Table S1).

These findings were corroborated using three novel *KRAS*^*G12C*^-mutant PDX models generated at a single institution from patients with *KRAS*^*G12C*^-mutant NSCLC who were at critical junctures in the current standard treatment care algorithm. Specifically, the models were generated from tumor biopsies taken from (i) a systemic treatment-naïve patient (PDX model NBC-NSCLC-M17), (ii) a patient at point of ICB resistance (PDX model NBC-NSCLC-KRAS3), and (iii) a patient resistant to both the first-line ICB and the second-line KRAS^G12C^(OFF) inhibitor sotorasib (PDX model NBC-NSCLC-KRAS1). These models were treated for 4 weeks with sotorasib, a RAS(ON) G12C-selective inhibitor (RMC-4998; ref. 11), a RAS(ON) multi-selective inhibitor (RMC-7977; ref. 16), or the RAS(ON) inhibitor doublet, followed by treatment cessation to assess durability of response. Consistent with the results described above for the well-established *KRAS*^*G12C*^ xenograft models (Fig. 1A and B; Supplementary Fig. S1C), in all cases, the RAS(ON) inhibitor doublet exhibited greater antitumor activity than each of the single agents (Fig. 1C). As anticipated, the NBC-NSCLC-KRAS1 PDX model was refractory to KRAS^G12C^(OFF) inhibitor monotherapy and also exhibited cross-resistance to RAS(ON) G12C-selective inhibitor monotherapy (Fig. 1C). Of note, daraxonrasib alone drove tumor stasis in this KRAS^G12C^ inhibitor–refractory model, and the RAS(ON) inhibitor doublet conferred even greater benefit than daraxonrasib alone in this model, with evidence of durable tumor growth inhibition upon treatment cessation (Fig. 1C). All tested treatment regimens were tolerated based on animal body weight assessment (Supplementary Fig. S1D and S1E).

### The RAS(ON) Inhibitor Doublet Forestalls Resistance Driven by Elevated RAS Pathway Flux

Hyperactivation of the RAS pathway and increased oncogenic flux are often associated with resistance to RAS inhibition (17). We hypothesized that the combinatorial effect of the RAS(ON) inhibitor doublet, particularly in the context of refractory disease, is a function of deeper suppression of the oncogenic RAS signaling driven by mutant RAS and/or WT RAS. We first established a role for elevated RAS signaling in two representative CDX (NCI-H2122 and NCI-H2030) and two PDX (CTG-2536 and LUN055) models prior to conduct of a detailed characterization of the activity of the RAS(ON) inhibitor doublet and respective monotherapies.

**Figure 2.**
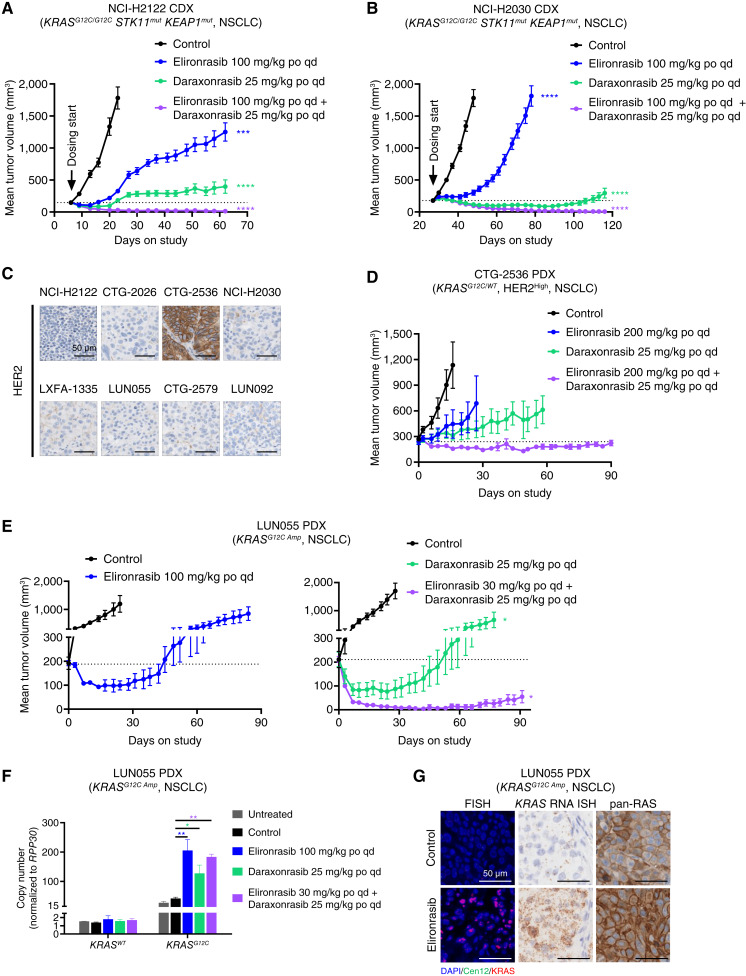
RAS(ON) inhibitor doublet forestalls resistance driven by elevated RAS pathway flux. **A,** Antitumor activity of elironrasib and daraxonrasib as single agents or in combination in the NCI-H2122 (*KRAS*^*G12C/G12C*^*STK11*^*mut*^*KEAP1*^*mut*^, NSCLC) subcutaneous xenograft model (*n* = 8–15 per group). Tumor-bearing mice were treated with vehicle or RAS(ON) inhibitors (elironrasib at 100 mg/kg orally every day and daraxonrasib at 25 mg/kg orally every day as single agents or in combination) for 17 to 56 days. Mean tumor volumes of each group were plotted over the course of treatment. Vehicle control and specific treatment groups were compared by two-way repeated-measures ANOVA on the last measurement day of the vehicle group (***, *P* < 0.001; ****, *P* < 0.0001). The dotted line indicates the initial average tumor volume. Error bars, SEM. **B,** Antitumor activity of elironrasib and daraxonrasib as single agents or in combination in NCI-H2030 (*KRAS*^*G12C/G12C*^*STK11*^*mut*^*KEAP1*^*mut*^, NSCLC) subcutaneous xenograft model (*n* = 8 per group). Tumor-bearing mice were treated with vehicle or RAS(ON) inhibitors (elironrasib at 100 mg/kg orally every day and daraxonrasib at 25 mg/kg orally every day as single agents or in combination) for 21–89 days. Mean tumor volumes of each group were plotted over the course of treatment. Vehicle control and specific treatment groups were compared by two-way repeated-measures ANOVA on the last measurement day of the vehicle group (****, *P* < 0.0001). The dotted line indicates the initial average tumor volume. Error bars, SEM. **C,** Histopathology analysis of HER2 in eight *KRAS*^*G12C*^-mutant NSCLC subcutaneous xenograft tumors at baseline included in the tumor response waterfall plot and Kaplan–Meier analysis in Fig. 1. Representative images are shown at 60× magnification. Scale bars, 50 μm. **D,** Antitumor activity of elironrasib, daraxonrasib, and elironrasib plus daraxonrasib combination in CTG-2536 (*KRAS*^*G12C/WT*^, HER2^High^, NSCLC) subcutaneous PDX model (*n* = 4–6 per group). Tumor-bearing mice were treated with vehicle or RAS(ON) inhibitors (elironrasib at 200 mg/kg orally every day, daraxonrasib at 25 mg/kg orally every day, and combination of elironrasib at 200 mg/kg orally every day plus daraxonrasib at 25 mg/kg orally every day) for 16–90 days. Mean tumor volumes of each group were plotted over the course of treatment. Vehicle control and specific treatment groups were compared by two-way repeated-measures ANOVA on the last measurement day of the vehicle group, and none of the comparisons were statistically significant. The dotted line indicates the initial average tumor volume. Error bars, SEM. **E,** Antitumor activity of elironrasib, daraxonrasib, and elironrasib plus daraxonrasib combination in the LUN055 (*KRAS*^*G12C Amp*^, NSCLC) subcutaneous PDX model (*n* = 3 per group). Tumor-bearing mice were treated with vehicle or RAS(ON) inhibitors (elironrasib at 100 mg/kg orally every day, daraxonrasib at 25 mg/kg orally every day and combination of elironrasib at 30 mg/kg orally every day plus daraxonrasib at 25 mg/kg orally every day) for 21–91 days. Mean tumor volumes of each group were plotted over the course of treatment. Vehicle control and specific treatment groups were compared by two-way repeated-measures ANOVA on the last measurement day of the vehicle group (*, *P* < 0.05). The dotted line indicates the initial average tumor volume. Error bars, SEM. Two tumor volume plots were from two different studies. **F,** Relative copy numbers of *KRAS*^*WT*^ or *KRAS*^*G12C*^ in LUN055 (*KRAS*^*G12C Amp*^, NSCLC) subcutaneous PDX tumors (*n* = 2–3 per group) collected at the end of tumor growth studies as shown in **E**. Tumors used for ddPCR analysis were collected at 3 hours after the last dose of vehicle or inhibitors. The relative gene copy numbers were determined by ddPCR and normalized to *RPP30*. Specific treatment group was compared with vehicle control using one-way ANOVA followed by Dunnett multiple comparison test (*, *P* < 0.05; **, *P* < 0.01). **G,** DNA FISH, RNA ISH, and histopathology analysis of LUN055 (*KRAS*^*G12C Amp*^, NSCLC) subcutaneous PDX tumors collected at the end of tumor growth studies as shown in **E**. Representative images are shown at 63×, 40×, and 40× magnification from ROIs closest to the mean of the group for FISH, RNA ISH, and IHC, respectively. Tumors used for representative images were collected at 3 hours after the last dose of vehicle or elironrasib. Scale bars, 50 μm. Cen12, centromere 12; ROI, region of interest; po, orally; qd, every day.

In the NCI-H2122 and NCI-H2030 CDX models, both of which represent the *KEAP1*- and *STK11*-deficient subset of NSCLC, elironrasib or daraxonrasib treatment alone induced initial tumor regressions followed by on-treatment regrowth (Fig. 2A and B). In contrast, the RAS(ON) inhibitor doublet drove durable complete tumor regressions (Fig. 2A and B). Similar observations were made *in vitro*, wherein the doublet resulted in more sustained RAS pathway inhibition compared with monotherapies at 48 hours after treatment in both cell lines, as measured by the levels of phosphorylation of ERK (pERK; Thr202/Tyr204) and P90RSK (pP90RSK; Thr359), two key downstream signaling components (Supplementary Fig. S2A and S2B). Highlighting a critical role for WT RAS proteins in maintaining pathway flux in NCI-H2030 cells following RAS(ON) mutant-selective inhibitor treatment, we demonstrated that *in vitro* elironrasib exhibited greater and more durable RAS pathway inhibition in cells with CRISPR-mediated knockout (KO) of *HRAS* and *NRAS* compared with the parental cell line (Supplementary Fig. S2C). In contrast, minimal differences in the inhibitory activity of the RAS(ON) multi-selective inhibitor daraxonrasib were observed between KO and parental cells (Supplementary Fig. S2C). Collectively, these data corroborate similar findings for preclinical tool RAS(ON) inhibitors in a genetically engineered mouse model (GEMM)-derived resistant model (8).

Upstream RTK activation can promote both KRAS^G12C^ and WT RAS GTP loading, increasing RAS pathway flux by mechanisms which are predicted to enhance sensitivity to the RAS(ON) inhibitor doublet (39). We used RTK overexpression as a surrogate for RTK activation in each of the NSCLC xenograft models shown in Fig. 1A and found that the CTG-2536 PDX model exhibited high baseline levels of HER2 mRNA and protein as assessed by RNA sequencing (RNA-seq) and immunohistochemistry (IHC) analyses, respectively (Supplementary Fig. S2D and S2E; Fig. 2C; Supplementary Table S3) relative to the other models. In this model, we observed tumor growth inhibition following single-agent RAS(ON) inhibitor treatments, whereas the doublet drove durable tumor regressions for the duration of the study (up to 90 days, Fig. 2D). *In vitro* studies confirmed that overexpression of HER2 or oncogenic RET M918T in NCI-H358 cells reduced elironrasib antiproliferative potency, as assessed by 3D CellTiter-Glo (CTG) assay, compared with the parental cells but did not influence the activity of the RAS(ON) inhibitor doublet (Supplementary Fig. S2F). Collectively, these results underscore the potential of a RAS(ON) inhibitor doublet to overcome RTK-driven pathway hyperactivation and resistance to RAS-targeted monotherapies.

The LUN055 NSCLC PDX model was chosen to explore the impact of *KRAS*^*G12C*^ amplification on RAS(ON) inhibition (*KRAS*^*G12C*^ gene copy number = 25; Supplementary Fig. S1B; ref. 13). In this model, elironrasib and daraxonrasib monotherapies drove initial tumor regressions but eventually showed on-treatment relapses (Fig. 2E). Interestingly, following long-term RAS(ON) inhibitor monotherapy treatment, we observed a further increase in *KRAS*^*G12C*^ gene copy number (as compared with controls; Fig. 2F). We verified the baseline as well as treatment-induced *KRAS* amplification in these tumors using fluorescence *in situ* hybridization (FISH; Fig. 2G). Similarly, elevated levels of *KRAS* mRNA and RAS protein were apparent following long-term dosing with elironrasib relative to controls (Fig. 2G). The RAS(ON) inhibitor doublet induced deep and durable tumor regressions in the LUN055 PDX model up to 90 days of treatment, demonstrating the potential to forestall therapeutic resistance through mutant *KRAS* amplification/overexpression (Fig. 2E). Two of three tumors in the RAS(ON) inhibitor doublet group started to relapse toward the end of the 90-day treatment period. The ddPCR analysis in these end-of-study tumors revealed a similar increase of *KRAS*^*G12C*^ gene copy number to that in the end-of-study tumors of monotherapy groups (Fig. 2F). *In vitro*, elironrasib exhibited reduced antiproliferative potency in NCI-H358 cells overexpressing exogenous KRAS^G12C^, as assessed by 2D CTG, compared with the control cells without KRAS^G12C^ overexpression, whereas the RAS(ON) inhibitor doublet provided a strong antiproliferative effect (Supplementary Fig. S2G).

### The RAS(ON) Inhibitor Doublet Drives Sustained RAS Pathway Suppression, Inhibits Cell Proliferation, and Induces Apoptosis *In Vivo*

**Figure 3.**
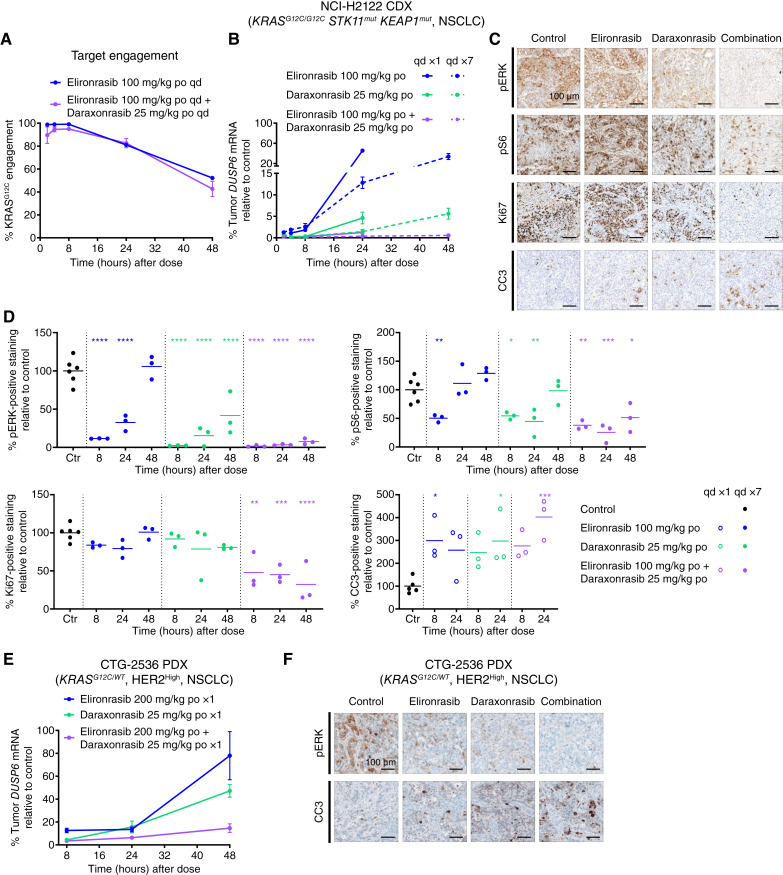
RAS(ON) inhibitor doublet drives sustained RAS signaling suppression, inhibits cell proliferation, and induces apoptosis *in vivo*. **A,** Target engagement of KRAS^G12C^ protein by elironrasib in NCI-H2122 (*KRAS*^*G12C/G12C*^*STK11*^*mut*^*KEAP1*^*mut*^, NSCLC) subcutaneous xenograft tumors, shown as the percentage of cross-linked KRAS^G12C^ by elironrasib relative to controls. Tumor-bearing mice were treated with 17 consecutive daily doses of elironrasib at 100 mg/kg as single agent (blue line) or in combination with daraxonrasib at 25 mg/kg (purple line). Tumors were harvested at indicated time points (*n* = 2–3 per time point). Values are plotted as mean ± SEM. **B,** PD in NCI-H2122 (*KRAS*^*G12C/G12C*^*STK11*^*mut*^*KEAP1*^*mut*^, NSCLC) subcutaneous xenograft tumors, shown as the relative change in human *DUSP6* mRNA expression. Tumor-bearing mice were treated with a single dose (every day ×1, solid lines) or seven consecutive daily doses (every day ×7, dashed lines) of elironrasib at 100 mg/kg and daraxonrasib at 25 mg/kg as single agents or in combination. Tumors were harvested at indicated time points (*n* = 2–3 per time point). Data from elironrasib single agent, daraxonrasib single agent, and RAS(ON) inhibitor doublet groups are plotted in blue lines, green lines, and purple lines, respectively. Values are plotted as mean ± SEM. **C** and **D,** Histopathology analysis of NCI-H2122 subcutaneous xenograft tumors treated with a single dose (every day ×1, circles in the quantification plot) or seven consecutive daily doses (every day ×7, dots in the quantification plot) of elironrasib at 100 mg/kg and daraxonrasib at 25 mg/kg as single agents or in combination and collected at indicated time points (*n* = 3 per time point). **C,** Representative images are shown at 20× magnification from samples closest to the mean of the group. Tumors used for representative images of pERK, pS6, and Ki67 were collected at 48 hours after seven consecutive daily doses of RAS(ON) inhibitors. Tumors used for representative images of CC3 were collected at 24 hours after a single dose of RAS(ON) inhibitors. Scale bars, 100 μm. **D,** pERK, pS6, and CC3 staining in tumor areas and Ki67 staining in tumor cell nucleuses were quantified and compared with vehicle using one-way ANOVA followed by Dunnett multiple comparison test (*, *P* < 0.05; **, *P* < 0.01; ***, *P* < 0.001; ****, *P* < 0.0001). **E,** PD in CTG-2536 (*KRAS*^*G12C/WT*^, HER2^High^, NSCLC) subcutaneous PDX tumors, shown as the relative change in human *DUSP6* mRNA expression. Tumor-bearing mice were treated with a single dose of elironrasib at 200 mg/kg and daraxonrasib at 25 mg/kg as single agents or in combination. Tumors were harvested at indicated time points (*n* = 2–3 per time point). Data from elironrasib single agent, daraxonrasib single agent, and RAS(ON) inhibitor doublet groups are plotted in blue lines, green lines, and purple lines, respectively. Values are plotted as mean ± SEM. **F,** Histopathology analysis of CTG-2536 subcutaneous PDX tumors treated with a single dose of elironrasib at 200 mg/kg and daraxonrasib at 25 mg/kg as single agents or in combination and collected at indicated time points (*n* = 3 per time point). Representative images are shown at 20× magnification from samples closest to the mean of the group. Tumors used for representative images of pERK and CC3 were collected at 48 and 24 hours after a single dose of RAS(ON) inhibitors, respectively. Scale bars, 100 μm. po, orally; qd, every day.

To explore the mechanism(s) driving the combinatorial benefit of the RAS(ON) inhibitor doublet, we profiled compound PK, KRAS^G12C^ TE, and RAS pathway PD *in vivo*. As shown in Supplementary Fig. S3A, the systemic exposures of elironrasib and daraxonrasib were consistent following single or repeated oral administration and comparable when administrated as single agents or in combination (Supplementary Table S4). We then examined KRAS^G12C^ TE by elironrasib, measured by the percentage of cross-linked KRAS^G12C^ protein in xenograft tumor cells using targeted liquid chromatography–tandem mass spectrometry (LC-MS/MS). Near-complete (>99%) TE was achieved at 8 hours after dose upon repeated administration of elironrasib at 100 mg/kg in NCI-H2122 xenograft tumors and decreased to 81% and 52% at 24 and 48 hours after the last dose of elironrasib, respectively (Fig. 3A; Supplementary Table S5). Coadministration with daraxonrasib did not affect KRAS^G12C^ TE by elironrasib *in vivo* (Fig. 3A; Supplementary Table S5), consistent with our observations *in vitro* wherein the levels of covalently modified KRAS^G12C^ were visualized using pan-RAS Western blot analysis (Supplementary Fig. S2A and S2B). Comparably high levels of KRAS^G12C^ TE were also observed in NCI-H358 xenograft tumors *in vivo* upon repeated administration of elironrasib as monotherapy or in combination with daraxonrasib (Supplementary Fig. S3B; Supplementary Table S5).

Consistent with the efficient KRAS^G12C^ TE in NCI-H2122 xenograft tumors, elironrasib induced >95% inhibition (relative to controls) of tumor RAS pathway signaling as assessed by downregulation of human *DUSP6* mRNA expression (Fig. 3B; Supplementary Table S5) for up to 8 hours after dose. Daraxonrasib alone induced somewhat deeper and more durable RAS pathway suppression than elironrasib alone, whereas the RAS(ON) inhibitor doublet exhibited similarly deep but more durable RAS pathway inhibition, e.g., maintaining >99% *DUSP6* inhibition (Fig. 3B; Supplementary Table S5) and sustained suppression on pERK and pS6 protein levels (Fig. 3C and D; Supplementary Table S3) for up to 48 hours after 7 days of treatment. Furthermore, transcriptomic analyses of RAS/MAPK pathway signatures also demonstrated deeper and more sustained inhibition in the RAS(ON) inhibitor doublet group compared with the respective monotherapies (Supplementary Fig. S3C; see “Methods” for details). Notably, RAS pathway inhibition at steady state for all RAS(ON) inhibitor treatments was generally comparable with that observed following single-dose administration (Fig. 3B; Supplementary Table S5). In addition, tumor cell proliferation, as assessed by Ki67 positivity, was significantly suppressed at all tested time points following a 7-day coadministration of elironrasib and daraxonrasib compared with controls (Fig. 3C and D; Supplementary Table S3), consistent with the observed decrease in G_2_M score in transcriptomic analyses (Supplementary Fig. S3C). Finally, both monotherapy and combination treatments significantly increased apoptosis (by 2–4-fold compared with controls) at 8 or 24 hours after dose, as assessed by the levels of cleaved caspase 3 (CC3) in tumors (Fig. 3C and D; Supplementary Table S3).

Similar combinatorial benefits with respect to sustained RAS pathway inhibition and increased induction of apoptosis with the RAS(ON) inhibitor doublet, relative to the monotherapies, were also observed in NCI-H2030 and CTG-2536 xenograft tumors (Fig. 3E and F; Supplementary Fig. S3D–S3G; Supplementary Tables S3 and S5).

### PK/TE/PD Modeling of Complementary Mechanisms Predicts the Superiority of the RAS(ON) Inhibitor Doublet over Monotherapies in the Context of KRAS^G12C^ Overexpression

**Figure 4.**
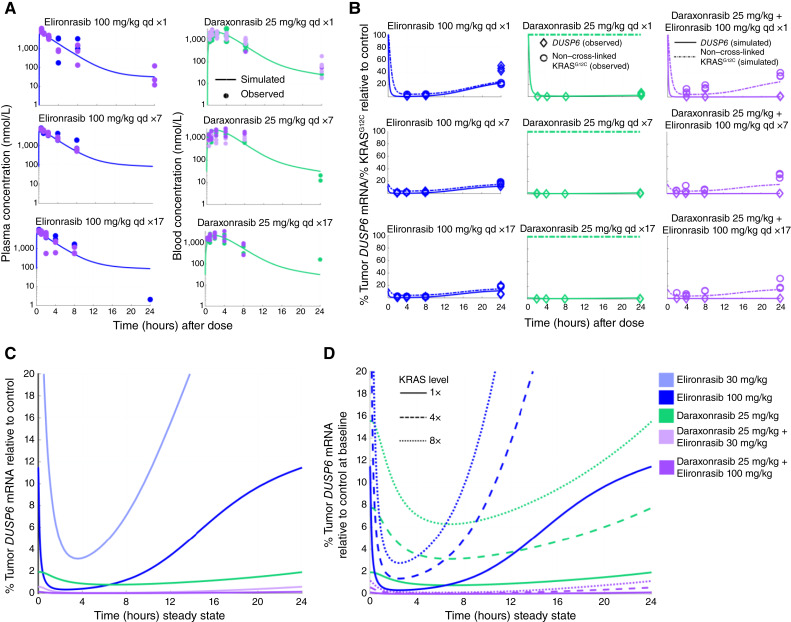
PK/TE/PD modeling of complementary mechanisms predicts the superiority of the RAS(ON) inhibitor doublet over monotherapies in the context of KRAS^G12C^ overexpression. **A,** The model captures the blood PK of daraxonrasib at 25 mg/kg and plasma PK of elironrasib at 100 mg/kg upon single and repeated daily administration. The simulated PK is shown by the solid lines. The observed data are shown by dots. **B,** Model captures the TE and PD upon single and repeated daily administration of elironrasib at 100 mg/kg and daraxonrasib at 25 mg/kg as single agents or in combination, as measured by the levels of non–cross-linked KRAS^G12C^ protein and *DUSP6* mRNA expression, respectively. Simulated TE and PD are shown by the dash and solid lines, respectively. The observed TE and PD data are shown by circles and diamonds, respectively. **C,** The steady-state simulation of RAS pathway suppression as measured by *DUSP6* mRNA expression relative to the baseline following daily administration of elironrasib at 30 or 100 mg/kg and daraxonrasib at 25 mg/kg as single agents or in combination. **D,** The steady-state simulation of RAS pathway suppression with different levels of KRAS^G12C^ protein as measured by *DUSP6* mRNA expression relative to the baseline without KRAS^G12C^ overexpression, following daily administration of elironrasib at 30 or 100 mg/kg and daraxonrasib at 25 mg/kg as single agents or in combination. qd, every day.

We next developed a mathematical PK/TE/PD model to relate circulating blood concentrations of daraxonrasib and plasma concentrations of elironrasib to the respective tumor concentrations, as well as to KRAS^G12C^ TE and *DUSP6* mRNA expression. The model incorporates known mechanisms of action on KRAS^G12C^, wherein elironrasib alone undergoes rapid covalent cross-linking with KRAS^G12C^ and noncovalent binding to free KRAS is expected to be a minor component, whereas daraxonrasib noncovalently binds to free KRAS^G12C^. Following elironrasib treatment, KRAS^G12C^ remains suppressed until non–cross-linked protein can be newly synthesized, whereas the effect of daraxonrasib on KRAS^G12C^ requires sustained concentrations in the tumor compartment. A graphical representation of the model and the species used for data fitting can be found in Supplementary Fig. S4A. The model was developed using single- and repeat-dose data collected from mice bearing NCI-H2122 xenograft tumors and was able to recapitulate the observed PK data (Fig. 4A) and PD/TE data (Fig. 4B) in terms of the initial drop, depth of inhibition, and trend toward the baseline. Notably, the simulated coadministration of both compounds resulted in deeper suppression of *DUSP6* mRNA than observed for either monotherapy at steady state across the dosing period (Fig. 4C), consistent with prior experimental observations.

As discussed above, *KRAS*^*G12C*^ amplification has been reported as a mechanism of clinical resistance to RAS inhibition (17, 18, 40). It has also been previously reported that there is a strong correlation between *KRAS* copy number and gene expression (41, 42), e.g., *KRAS* amplification is associated with a significant increase in protein expression in the NSCLC cell line cohort from the Cancer Cell Line Encyclopedia (accessed through cBioPortal; Supplementary Fig. S4B; refs. 43–45). Thus, to predict the impact of *KRAS*^*G12C*^ amplification on RAS pathway suppression following RAS(ON) inhibitor treatment, we simulated up to eightfold overexpression of KRAS^G12C^ protein by increasing the synthesis rate in the model. Upon KRAS^G12C^ overexpression, elironrasib single-agent treatment exhibited an accelerated rebound in free KRAS^G12C^ and *DUSP6* mRNA expression as compared with the baseline model. In contrast, daraxonrasib single-agent treatment maintained durable RAS pathway inhibition, due to prolonged tumor retention, albeit the depth of pathway inhibition was reduced in the context of KRAS^G12C^ overexpression. Indeed, neither single agent could maintain >90% reduction in *DUSP6* mRNA upon an eightfold increase in the steady-state level of KRAS^G12C^ protein levels, whereas the RAS(ON) inhibitor doublet was predicted to maintain ≥98% *DUSP6* inhibition across the dosing interval (Fig. 4D). These conclusions are consistent with the experimental observations in the LUN055 model described above.

### Daraxonrasib, Elironrasib, and the RAS(ON) Inhibitor Doublet Drive Immune-Dependent Tumor Clearance in Mice and Synergize with Anti–PD-1 in an Immunogenic NSCLC Model

Oncogenic KRAS plays a significant role in dampening antitumor immunity. Prior studies have shown that RAS inhibition in cancer cells can increase antitumor immunity and synergize with checkpoint inhibitors in immune-infiltrated lung tumor models, supporting its translational potential in patients with NSCLC (46). In this study, we investigated whether deep RAS pathway suppression via RAS(ON) inhibitor doublet therapy could maximize antitumor immunity and improve responses to anti–PD-1.

**Figure 5.**
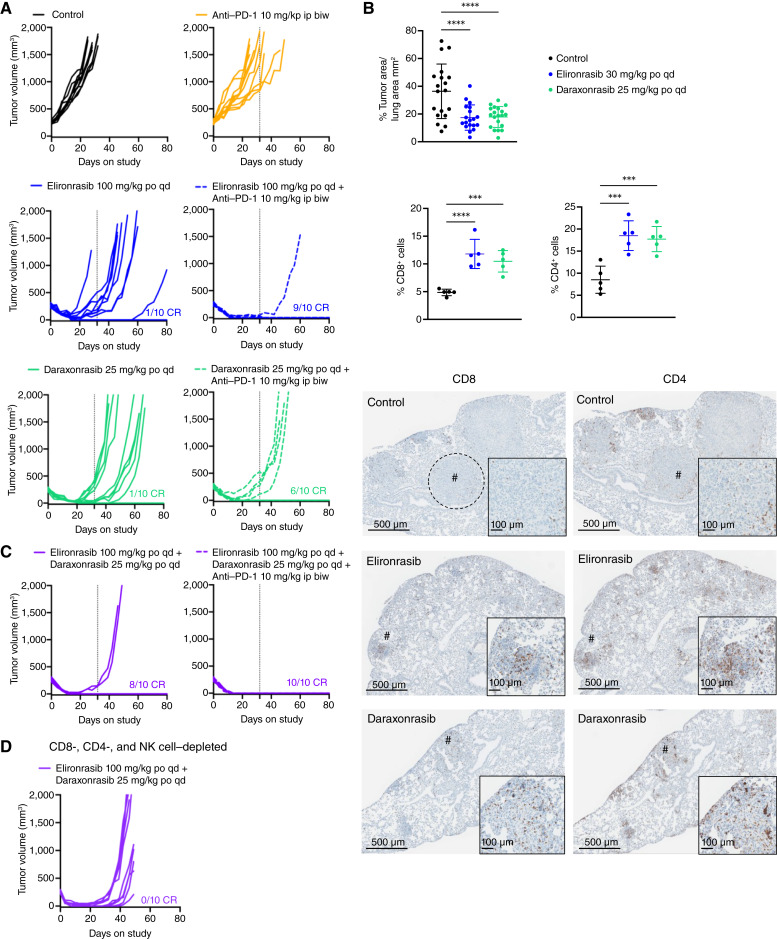
Elironrasib and daraxonrasib drive immune-dependent CRs, favorably modulate the TME, and synergize with anti–PD-1 in the immunogenic KPAR1.3 model. **A,** Tumor growth of subcutaneous tumors treated for 32 days (dashed line indicated treatment stop) with vehicle, elironrasib at 100 mg/kg, daraxonrasib at 25 mg/kg orally every day in combination with mouse IgG2a (isotype control), or anti–PD-1 at 10 mg/kg intraperitoneally twice a week in C57BL/6 mice shown as individual tumor volume (*n* = 10). Number of CRs indicated. **B,** Representative lung IHC images (large image – scale bar, 500 μm; zoomed in image – scale bar, 100 μm; # denotes enlarged area; dashed lines highlight large tumor nodules), percentage tumor area per lung area at 4 hours after 4 days of treatment with elironrasib at 30 mg/kg or daraxonrasib at 25 mg/kg orally every day (four lung sections were evaluated for each mouse) and quantification of CD8^+^ or CD4^+^ T cells as percentage of all cells within the tumor region in orthotopic lung tumors. Each point is the analysis of the tumor mass/section of lung. Four lung sections were evaluated for each mouse. Asterisks indicate analysis was performed using one-way ANOVA. For all statistical analysis ***, *P* < 0.001; ****, *P* < 0.0001. **C,** Tumor growth of subcutaneous tumors treated for 32 days (dashed line indicated treatment stop) with elironrasib at 100 mg/kg in combination with daraxonrasib at 25 mg/kg orally every day and mouse IgG2a (isotype control) or anti–PD-1 at 10 mg/kg intraperitoneally twice a week in C57BL/6 mice shown as individual tumor volume (*n* = 10). **D,** Tumor growth of subcutaneous tumors treated for 32 days with elironrasib at 100 mg/kg or daraxonrasib at 25 mg/kg orally every day in the presence of CD4 and CD8 T cell–depleting and NK cell–depleting antibodies administered at 10 mg/kg at day -1, 0, and every 6 days after dosing start. CRs are defined as tumor volume at 0 mm^3^. po, orally; qd, every day; biw, twice a week.

Given that RAS(ON) multi-selective inhibitors, like daraxonrasib, inhibit WT RAS in immune cells (16), we first evaluated daraxonrasib activity, alone and in combination with anti–PD-1, as compared with RAS(ON) G12C-selective inhibition via elironrasib in the immunogenic KPAR1.3 GEMM-derived syngeneic NSCLC model harboring the *Kras*^*G12C*^ mutation. This model is T cell–infiltrated and partially responsive to immunotherapy due to the expression of a derepressed endogenous retroviral antigen (47). As expected, anti–PD-1 monotherapy delayed tumor growth. Daraxonrasib as a single agent achieved durable complete regressions (CR) in 10% (1/10) of mice and demonstrated notable synergy in combination with anti–PD-1, resulting in 60% (6/10) durable CR. Elironrasib treatment showed comparable activity to daraxonrasib with 10% (1/10) CR as a single agent and synergy [90% (9/10) CR] in combination with anti–PD-1 (Fig. 5A; Supplementary Fig. S5A and S5B). Next, we assessed whether RAS (ON) inhibition altered immune cell infiltration in KPAR1.3 orthotopic lung tumors. The lungs from vehicle control–treated mice showed a mixture of small, immune-infiltrated tumors and large tumor nodules that were mostly CD4 and CD8 T cell–excluded. In contrast, mice treated with daraxonrasib or elironrasib for 4 days showed a significant reduction in tumor mass, characterized by the lack of well-defined nodular structures and by a high infiltration with CD4 and CD8 T cells (Fig. 5B). To assess whether the cell death induced by RAS(ON) inhibitors enhances immunogenicity, we measured the expression of immunogenic cell death (ICD) hallmarks. *In vitro*, both elironrasib and daraxonrasib increased (to a similar extent) markers of ICD, such as HMGB-1 release and calreticulin externalization, to levels comparable with the known ICD inducer mitoxantrone (MTX; Supplementary Fig. S5C). To confirm the induction of ICD *in vivo*, we vaccinated mice subcutaneously with KPAR1.3 cells treated with daraxonrasib, elironrasib, or MTX *in vitro* and challenged the mice with live KPAR1.3 cells after 1 week on the contralateral side. Vaccination with daraxonrasib- or elironrasib-treated cancer cells delayed tumor growth *in vivo*, comparable with MTX (Supplementary Fig. S5D), suggesting that RAS(ON) inhibition can enhance tumor immunogenicity. Overall, these data suggest that in addition to suppressing cancer cell proliferation, daraxonrasib has the potential to increase T-cell infiltration and induce ICD, thus priming tumors for immunotherapy and synergy with anti–PD-1. Notably, the antitumor immunity induced by daraxonrasib is comparable with that observed with elironrasib, which selectively inhibits oncogenic RAS^G12C^(ON).

Next, we interrogated the activity of the doublet combination of daraxonrasib with elironrasib and a triplet including anti–PD-1 in the KPAR1.3 model. As observed in multiple xenograft models described above, the RAS(ON) inhibitor doublet drove deeper and more durable responses compared with monotherapies in this model, achieving 80% (8/10) CR, compared with 10% (1/10) with single agents (Fig. 5C; Supplementary Fig. S5A). The triplet combination of daraxonrasib, elironrasib, and anti–PD-1 prevented tumor relapse after treatment withdrawal in all mice (10/10; Fig. 5C; Supplementary Fig. S5A and S5B). To evaluate the extent to which the adaptive immune system is required for the antitumor effects, we performed combined depletions of CD4^+^ T, CD8^+^ T, and NK cells prior to dosing with RAS(ON) inhibitors. Daraxonrasib, elironrasib, and the RAS(ON) inhibitor doublet all drove initial deep regressions, similar to the response in naïve mice. However, all tumors regrew upon dosing cessation in contrast to the CRs observed in naïve mice, indicating that tumor-infiltrating lymphocytes (TIL) are indispensable for the durability of response to RAS(ON) inhibition in this model (Fig. 5D; Supplementary Fig. S5E and S5F).

### The RAS(ON) Inhibitor Doublet Maximizes Pathway Suppression and Favorably Modulates the TME of an ICB-Refractory NSCLC Model

Despite the survival benefits of ICB in advanced NSCLC, a substantial subset of patients fails to respond, particularly those with poor T-cell infiltration (48–50) or tumors exhibiting MHC class I downregulation, a common mechanism of immune evasion in NSCLC (25). To model such an ICB-refractory NSCLC population, we utilized the Kras^G12C^ dependent 3LL-ΔNRAS mouse model that carries a *Cdkn2a/b* deletion, has a TME that is T cell–excluded, is dominated by immunosuppressive cells, and generally accepted as being refractory to pharmacologic intervention (30). In addition, despite high TMB (46 mut/mb), which in patients is a predictive biomarker for response to ICB therapies (51–53), the 3LL-ΔNRAS model exhibits suppressed MHC class I (H2-Kb) expression. Previous studies have shown that single-agent RAS^G12C^ mutant-selective inhibition is not sufficient to sensitize 3LL-ΔNRAS tumors to anti–PD-1 (46). We hypothesized that the deeper and more sustained RAS pathway suppression achieved with the RAS(ON) inhibitor doublet could reverse oncogenic RAS-driven immunosuppressive mechanisms and thereby prime the TME for effective combination with ICB.

**Figure 6.**
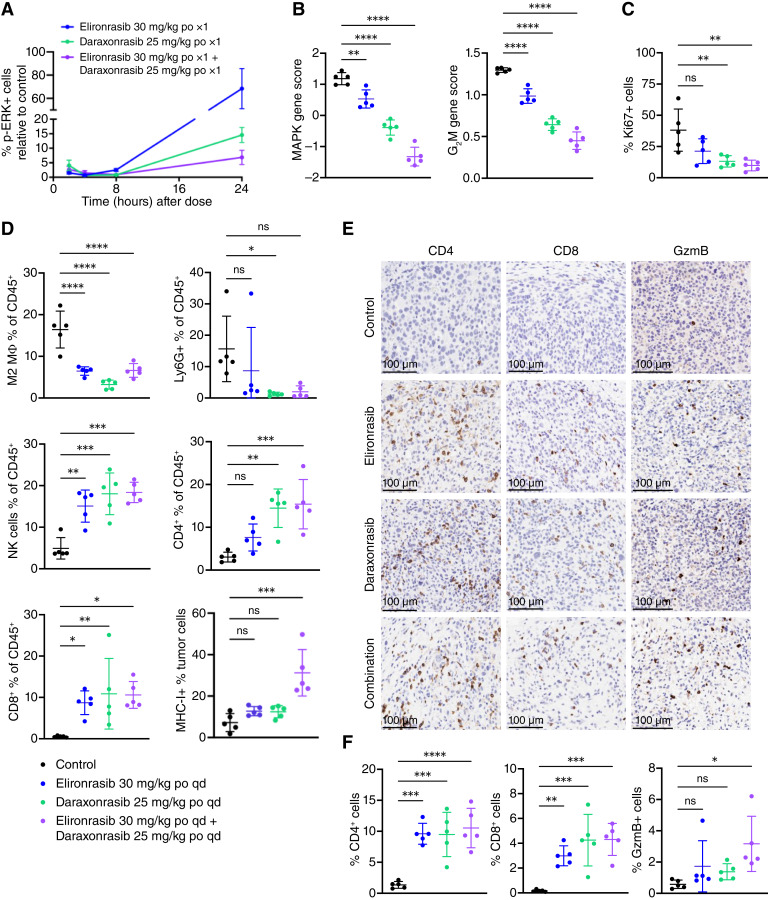
RAS(ON) doublet maximizes pathway suppression and favorably modulates the TME of the ICB-refractory 3LL-ΔNRAS model. **A,** PD of elironrasib, daraxonrasib, and the RAS(ON) doublet in subcutaneous tumors shown as the relative change in pERK expression in tumor cells. Tumor-bearing mice were treated with a single dose of elironrasib at 30 mg/kg, daraxonrasib at 25 mg/kg, and the RAS(ON) doublet and tumors were collected at indicated time points. **B,** PROGENy MAPK and G_2_M score calculated based on bulk RNA-seq of whole tumors collected at 24 hours after 8 days of treatment. **C,** Quantification of as percentage of total cells in tumors collected at 24 hours after 8 days of treatment. **D,** Frequency of CD8 T cells, CD4 T cells, NK cells, granulocytic myeloid-derived suppressor cells (gMDSC; Ly6G+), M2 macrophages (CD11b+, F4/80+, CD206+) based on live, CD45^+^ cells, and H2K^b^ cell surface expression on tumor cells (large CD45^−^, periostin−) identified by multiparameter spectral flow cytometry in tumors collected at 24 hours after 8 days of treatment. **E,** Representative IHC images (scale bar, 100 μm) and (**F**) Quantification of CD4-, CD8-, and granzyme B–positive cells as percentage of total in tumors collected at 24 hours after 8 days of treatment. Values are plotted as mean ± SD. Each point is an individual tumor. Analysis was performed using one-way ANOVA. For all statistical analyses, *, *P* < 0.05; **, *P* < 0.01; ***, *P* < 0.001; ****, *P* < 0.0001. po, orally; qd, every day.

First, we confirmed that the RAS(ON) inhibitor doublet maximized pathway suppression in this model comparably with xenografts, as assessed histologically by pERK expression in tumor cells at 24 hours after a single dose, as well as a 7-day repeat dose (Fig. 6A; Supplementary Fig. S6A–S6C) and by transcriptomic analysis in whole tumor tissue at 24 hours after 8 days of dosing (Fig. 6B). Additionally, the decrease in DNA replication represented by the G_2_M PROGENy score and proliferation evaluated by Ki67 IHC staining confirmed the increased depth of response to the RAS(ON) doublet (Fig. 6B and C; Supplementary Fig. S6D). This PD modulation in tumors was accompanied by a significant favorable modulation of the TME. During the acute response, at 24 hours after 8 days of dosing, daraxonrasib, elironrasib, and the RAS(ON) doublet all decreased tumor infiltration of immunosuppressive M2 macrophages and granulocytic myeloid-derived suppressor cells and increased tumor proportions of CD4^+^ and CD8^+^ T cells, as well as NK cells (Fig. 6D). Histologic analyses not only confirmed that the number of T cells in tumors was increased, as noted by flow cytometry, but also highlighted that this immune-excluded tumor model became infiltrated with TILs upon treatment (Fig. 6E and F). Consistent with reports that oncogenic KRAS can dampen IFNγ signaling, maximizing RAS pathway suppression increased the expression of IFNγ and its receptors in whole tumor tissue, highlighting a potential mechanism to reverse immune evasion and enhance cytotoxicity (Supplementary Fig. S6E). Although all treatment regimens had a similar impact on the TME composition, flow cytometric analysis of large CD45^−^, periostin− cells (tumor cells) showed that only the RAS(ON) doublet induced significant expression of MHC class I (H2-Kb) and GzmB IHC staining in tumors, indicating an increased cytotoxicity profile (Fig. 6D–F). This suggests that maximizing RAS(ON) inhibition in tumor cells leads to a reversal of the immunosuppressive program mediated by oncogenic KRAS^G12C^ and has the potential to render immune-excluded tumors sensitive to ICB.

### The RAS(ON) Inhibitor Doublet Maximizes Antigen Presentation and Sensitizes an ICB-Refractory Tumor Model of NSCLC to Anti–PD-1

**Figure 7.**
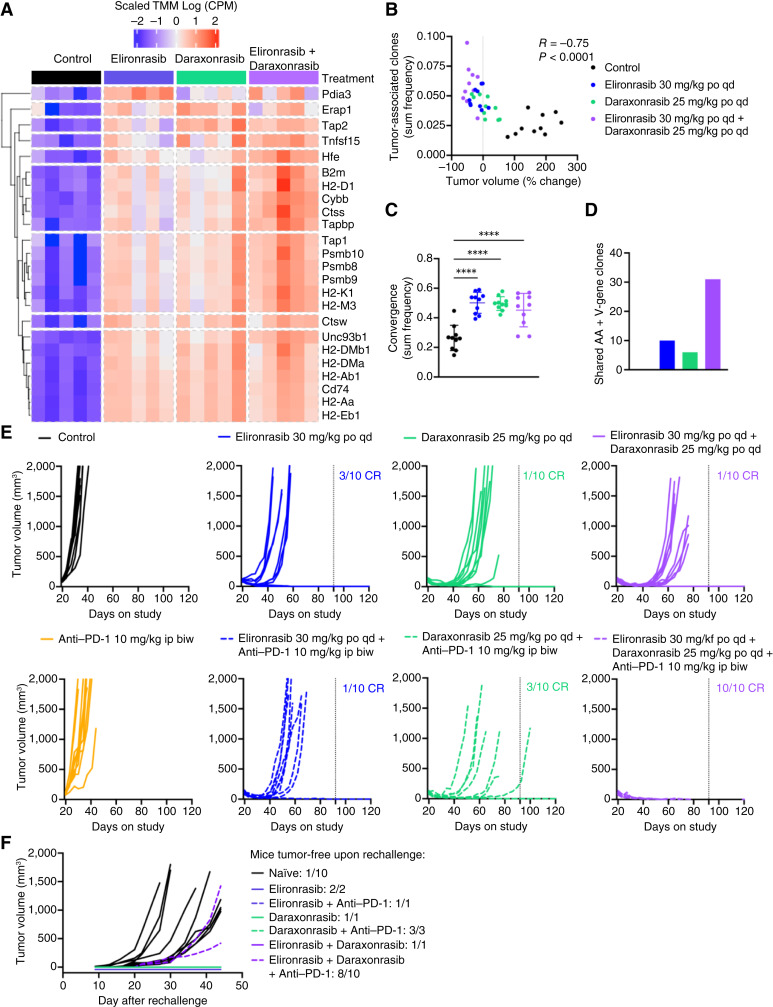
RAS(ON) doublet maximizes antigen presentation and sensitizes the ICB-refractory 3LL-ΔNRAS tumor model to anti–PD-1. **A,** Heat map of individual antigen presentation machinery signature genes. Differential expression in tumors (*n* = 5 per treatment) collected at 24 hours after 8 days of treatment with vehicle, elironrasib at 30 mg/kg, daraxonrasib at 25 mg/kg, or the RAS(ON) doublet. **B–D,** Immuno-sequencing (TCRB assay) of gDNA extracted from tumors collected at 24 hours after 8 days of treatment (**B**), Correlation of percent change in the tumor volume from predose to day 8 with the increase in T-cell abundance in the treated tumors. **C,** Sum frequency of convergent clones in the tumor. Analysis was performed using one-way ANOVA. For all statistical analyses ****, *P* < 0.0001. **D,** number of shared clones (amino acid + V-gene) within each treatment group that were found in each of the 10 tumors/group. **E,** Tumor growth of subcutaneous tumors treated for 72 days (dashed line represents treatment stop) with vehicle, elironrasib at 30 mg/kg, or daraxonrasib at 25 mg/kg orally every day in combination with mouse IgG2a (isotype control) or anti–PD-1 at 10 mg/kg intraperitoneally twice a week in C57BL/6 mice shown as individual tumor volume (*n* = 10). Number of CRs indicated. **F,** Tumor growth for the mice from (**E**) with durable CR for 76 days that were rechallenged on the opposite flank. Table indicates treatment that the primary tumor received and number of mice with immune rejections. Naïve mice were used as controls. CRs are defined as tumor volume at 0 mm^3^. po, orally; qd, every day; bid, twice a week.

Given the critical role of antigen presentation by cancer cells in enabling T cell–mediated recognition and cytotoxicity, and its relevance to ICB efficacy in NSCLC, we next assessed the impact of the RAS(ON) inhibitor doublet on the broader antigen presentation machinery at the transcriptomic level. Bulk RNA-seq of 3LL-ΔNRAS tumors, harvested 24 hours after 8 days of dosing, revealed that both elironrasib or daraxonrasib single agents increased the expression of antigen processing and presentation machinery genes, with the RAS(ON) inhibitor doublet achieving the strongest upregulation (Fig. 7A). Enhanced antigenicity was further supported by T-cell receptor (TCR) sequencing, which showed increased T-cell abundance across all treatments, most notably with the RAS(ON) inhibitor doublet (Supplementary Fig. S7A). This increase in T-cell infiltration (normalized by input DNA) correlated with tumor volume reduction at endpoint (Fig. 7B; Supplementary Fig. S7A). Although TCR clonality and richness remained unchanged across groups (Supplementary Fig. S7B and S7C), all treatments significantly increased the sum frequency of convergent clones (TCRs with shared antigen specificity) within the tumor (Fig. 7C). Convergent T-cell clones, previously shown to express CD8^+^ cytotoxic gene signatures in both human and murine TMEs (54), are indicative of antigen-specific immune engagement and have been associated with improved patient survival with ICB therapy (54). Finally, the number of shared TCR clones (by amino acid and V-gene) across tumors within each treatment group was markedly elevated in the RAS(ON) inhibitor doublet cohort compared with single agents, suggesting a broader and more focused T-cell response to shared tumor antigens (Fig. 7D).

Lastly, we sought to evaluate whether the reversal of the immunosuppressive TME and the boost in antigen presentation in response to RAS(ON) inhibitor doublet is sufficient to sensitize this refractory model to anti–PD-1. Although elironrasib and daraxonrasib as single agents drove 10% (1/10) and 30% (3/10) CR, respectively, most 3LL-ΔNRAS tumors relapsed on treatment (Fig. 7E). The RAS(ON) inhibitor doublet achieved deeper tumor regressions initially compared with single agents, but even in this regimen, the majority of the tumors relapsed (90%) after 50 days of treatment. Although we demonstrated that elironrasib and daraxonrasib caused a profound remodeling of the TME, in the absence of an increase in MHC class I, the single agents did not render these tumors sensitive to anti–PD-1 (Fig. 7E). However, by enhancing tumor recognition, the RAS(ON) inhibitor doublet could sensitize this ICB-refractory 3LL-ΔNRAS model to anti–PD-1, driving 100% (10/10) durable CR over the duration of the study (154 days, Fig. 7E).

To investigate whether RAS(ON) inhibition triggered systemic antitumor immunity and promoted immune memory in 3LL-ΔNRAS tumors, we rechallenged the tumor-free mice in each group approximatively 2 months after the dosing cessation on the contralateral flank in the absence of further treatment. Although 9 of 10 tumors grew in the naïve mice, no tumors grew in any of the rechallenged mice previously treated with the RAS(ON) single-agent inhibitors or the RAS(ON) inhibitor doublet. In the triplet group, 8 of 10 animals remained tumor-free. These data indicate that both elironrasib and daraxonrasib alone and in combination promote the induction of antitumor immune responses, leading to long-term protection from tumor recurrence (Fig. 7F).

Collectively, these preclinical findings suggest that the RAS(ON) doublet combination of RAS(ON) mutant-selective and multi-selective inhibition can achieve deeper pathway suppression, reverse the immune evasion mechanisms governed by oncogenic RAS, and sensitize ICB-refractory tumors to PD-L/1 blockade driving durable and complete tumor regressions in a triplet regimen.

## Discussion

The FDA approval of sotorasib (Lumakras) and adagrasib (Krazati) marks a significant advancement in targeted therapy treatments for *KRAS*^*G12C*^-mutant cancers, particularly NSCLC (3–7). However, these first-generation KRAS^G12C^(OFF) inhibitor monotherapies have demonstrated limited clinical efficacy due to primary and acquired resistance mechanisms, the majority of which involve the reactivation of the RAS pathway in tumors (17, 18, 55, 56). Therefore, a clear need exists for the development of more effective KRAS^G12C^ inhibitors, as well as combination strategies to improve treatment outcomes and overcome resistance (57–60). Multiple combinations of KRAS^G12C^ inhibitors with targeted therapies directed at both in- and parallel-pathway have been investigated (61, 62). In particular, the in-pathway combination of KRAS^G12C^ and EGFR inhibition in colorectal cancer has led to the recent approval of adagrasib plus cetuximab and sotorasib plus panitumumab combination therapies in metastatic *KRAS*^*G12C*^-mutant colorectal cancer (mCRC; refs. 63–65). These combination approaches highlight the potential for WT RAS inhibition (via EGFR blockade and vertical pathway inhibition in this case) to augment KRAS^G12C^ inhibition and achieve improved RAS pathway suppression to forestall resistance. In this study, we utilized a comprehensive set of preclinical models of *KRAS*^*G12C*^-mutant NSCLC that display reduced response and/or resistance to RAS^G12C^ inhibitor monotherapy, including a newly assembled patient-derived series from a single institution. Our findings demonstrate the benefit of “doubling down” on oncogenic RAS with concomitant inhibition of mutant and WT RAS(ON) with the novel RAS(ON) inhibitor doublet of elironrasib and daraxonrasib.

We have previously reported that the RAS(ON) G12C-selective covalent inhibitor elironrasib and the RAS(ON) multi-selective inhibitor daraxonrasib alone can overcome some of the limitations of the KRAS^G12C^(OFF) inhibitors (11, 13, 16). In this study, we now show that combination treatment with a RAS(ON) inhibitor doublet leads to durable RAS pathway suppression and, consequently, improved antitumor activity as compared with either monotherapy. Importantly, we demonstrate that the combination of elironrasib and daraxonrasib has greater potential (compared with either single agent) to avert resistance driven by elevated RAS pathway flux, e.g., RTK activation or *KRAS*^*G12C*^ amplification.

To better understand the relationship between covalent and noncovalent inhibition of KRAS^G12C^ by elironrasib and daraxonrasib, respectively, we established a mathematical model to relate systemic and tumor concentrations with KRAS^G12C^ TE and PD modulation of RAS signaling via *DUSP6* inhibition. The durability of RAS pathway suppression with an efficient covalent KRAS^G12C^ inhibitor, such as elironrasib, is mainly dependent on the time required to synthesize new KRAS^G12C^ protein in the tumor cells. Daraxonrasib suppresses RAS pathway signaling by reversibly targeting the KRAS^G12C^(ON) protein and sterically disrupting the binding to downstream effectors. Hence, durable pathway suppression by daraxonrasib was driven by the sustained steric occlusion of RAS(ON) protein enabled by its relatively long tumor retention time. Due to the complementary mechanisms of action of the two RAS(ON) inhibitors, upon combination, elironrasib rapidly and irreversibly cross-linked the KRAS^G12C^ protein, leaving a reduced pool of protein to be bound by daraxonrasib. Thus, the RAS(ON) inhibitor doublet is predicted to achieve deeper and more durable RAS pathway suppression compared with either monotherapy across the entirety of the dosing interval, even at suboptimal dose levels of the respective agents, in both baseline and *KRAS*^*G12C*^ amplification settings. Indeed, compared with monotherapies, the model predicts that the doublet advantage becomes increasingly significant with increasing levels of KRAS^G12C^. It should be noted that the modeling results presented here are based on short-term data from one xenograft model and that other factors, such as higher levels of KRAS protein expression and/or additional comutations, may influence the combination benefit in different models or in the clinic. Nevertheless, the modeling exercises underscore the potential to maximize oncogenic RAS signaling when combining two KRAS inhibitors with complementary mechanisms of actions.

A large body of evidence supports a key role for oncogenic RAS (and other oncogenes) in immunosuppression in cancers (66). Mutant KRAS is reported to decrease T-cell infiltration and downregulate antigen presentation, leading to reduced tumor sensitivity to immunotherapies (67).

In this study, we demonstrate that the RAS(ON) G12C-selective inhibitor, elironrasib synergized with ICB in an immunogenic syngeneic *Kras*^G12C^-mutant NSCLC model, consistent with previous reports (46). Furthermore, we show that the antitumor immune response elicited by daraxonrasib, including T-cell infiltration, immunologic memory induction, and its combinatorial benefit with anti–PD-1, is comparable with that of the RAS(ON) G12C-selective inhibitor. This observation is significant in that RAS(ON) multi-selective inhibitors, such as daraxonrasib, can target RAS WT in immune cells and inhibit naïve T-cell proliferation *in vitro*, but as shown previously do not affect proliferation in preactivated T cells *in vitro* or affect T-cell priming *in vivo* (13, 68). We further highlight the role of adaptive immunity in achieving durable CRs to the RAS(ON) inhibitor doublet treatment in these T cell–infiltrated tumors.

In a T cell–excluded NSCLC tumor, with suppressed MHC I expression, the combination of daraxonrasib with elironrasib maximized RAS pathway inhibition and drove robust increases in antigen presentation and T-cell infiltration. Corresponding TCR repertoire analysis revealed increased convergence and higher numbers of shared TCR clones across tumors, indicative of enhanced tumor antigen recognition by tumor-resident T cells. These findings support the ability of the RAS(ON) inhibitor doublet to reprogram the TME and sensitize ICB-refractory tumors to anti–PD-1 therapy. We propose that maximizing RAS pathway suppression in NSCLC may restore immunogenicity and expand the clinical benefit of immunotherapy to patients who are currently unresponsive. Notably, similar strategies, such as the combination of BRAF and MEK inhibitors with anti–PD-1, have shown encouraging activity in ICB-refractory *BRAF*^V600E^-mutant microsatellite-stable colorectal cancer (69). The increased antigen presentation and expanded TCR recognition observed in this study also provide a rationale for evaluating vaccine-based immunotherapies in combination with RAS(ON) inhibition. Finally, incorporating immunotherapy may also help eliminate emergent resistant clones following RAS inhibition.

The present study highlights the potential for a RAS(ON) inhibitor doublet comprised of elironrasib and daraxonrasib to deepen and prolong antitumor activity in particular in *KRAS*^*G12C*^-mutant NSCLC models refractory to monotherapy treatments. Although this study has focused on *KRAS*^*G12C*^-mutant NSCLC tumors, the experimental evidence and underlying mechanism suggest that this strategy may be generalizable to other mutant selective inhibitors and/or tumor types. Similarly, the potential for a RAS(ON) inhibitor doublet to abrogate key immune evasive mechanisms within the TME, and engender a therapeutic vulnerability to immunotherapy, may be relevant in other immunorefractory tumors. In sum, the encouraging preclinical results described herein provide strong scientific rationale for the evaluation of the RAS(ON) inhibitor doublet of elironrasib and daraxonrasib in combination with pembrolizumab in patients with *KRAS*^*G12C*^-mutant NSCLC (NCT06162221).

## Methods

### Cell Cultures and Reagents

NCI-H2122, NCI-H2030, and NCI-H358 cells were purchased from the ATCC and maintained *in vitro* as a monolayer culture in an appropriate medium supplemented with 10% fetal bovine serum (FBS). All cells were maintained at 37°C in a humidified incubator at 5% CO_2_. Cells in the exponential phase of growth were harvested for tumor cell inoculation. The NCI-H2122 cell line was obtained in April 2020 and authenticated in May 2024. The NCI-H2030 cell line was obtained in October 2019 and authenticated in May 2024. The NCI-H358 cell line was obtained in April 2020. The *CellCheck 16 Plus* platform from IDEXX BioAnalytics was used to authenticate human cell lines and screen for *Mycoplasma* contamination. All described *in vitro* experiments were performed within three passages after thawing. The 3LL NRAS KO (3LL-ΔNRAS) cell line is a murine Lewis lung adenocarcinoma engineered cell line with NRAS knocked out using CRISPR-Cas9. KPAR1.3 (KPAR1.3G12C) is derivative from the KPAR cells, in which the mutation Asp in position 12 from KRAS (G12D) was mutated to Cys (G12C) using prime editing. The 3LL NRAS KO cell line (cat. #157772) and the KPAR1.3G12C cell line (cat. #161804) were engineered by the Julian Downward Laboratory in the Crick Institute, United Kingdom, and are available through cancertools.org.

#### Cell Line Engineering

*H/NRAS* KO NCI-H2030 cells were generated using CRISPR technology with the Cas9:gRNA ribonucleoprotein delivery method. The multiguide RNA for generating *H/NRAS* gene KO was designed and synthesized at Synthego. A total of 2 × 10^5^ cells were harvested and resuspended in Resuspension Buffer R (Thermo Fisher Scientific, MPK1025) immediately prior to transfection. A volume of 50 pmol of TrueCut V2 Cas9 (Thermo Fisher Scientific, A36498), 75 pmol of *HRAS* multiguide RNA, and 75 pmol of *NRAS* multiguide RNA were mixed and incubated at room temperature for 10 minutes to form the Cas9:gRNA ribonucleoprotein complex. The Cas9:gRNA ribonucleoprotein complexes were added to the 2 × 10^5^ cells in Resuspension Buffer R to reach a final volume of 20 μL immediately prior to electroporation. Electroporation was then performed using the Neon Transfection System (Invitrogen, MPK5000). The following parameters were used for transfection: pulse voltage: 1550 V, pulse width: 20 milliseconds, and pulse number: 1. The transfected NCI-H2030 cells were incubated in prewarmed antibiotic-free media (RPMI + 20% FBS) for 3 days to recover before switching to complete media. Sequences of guide RNAs: Human *HRAS* (guide #1: 5′-GGAUUCCUACCGGAAGCAGG-3′; guide #2: 5′-GUGGAUGUCCUCAAAAGACU-3′; guide #3: 5′-GCAUGGCGCUGUACUCCUCC-3′), and Human *NRAS* (guide #1: 5′-GGAUUCUUACAGAAAACAAG-3′; guide #2: 5′-CCUCAUGUAUUGGUCUCUCA-3′; guide #3: 5′-CAAUAAUAGCAAGUCAUUUG-3′). *H/NRAS* KO NCI-H2030 cells were generated in March 2025 from the authenticated parental NCI-H2030 cell line.

#### Inducible Full-Length RTK and KRAS^G12C^ Overexpression NCI-H358 Cell Lines

Plasmids encoding the tet-controlled transcriptional silencer (tTS), reverse tet-controlled transcriptional activator (rtTA), and tet-inducible receptor tyrosine kinases (RTK) or KRAS^G12C^ were synthesized and packaged into lentivirus at Vector Builder. Lentivirus transductions were performed with the addition of polybrene (4 μg/mL). NCI-H358 cells were transduced with lentivirus encoding tTS/rtTA for 48 hours prior to the selection with blasticidin (5 μg/mL) for 12 days. The concentration of blasticidin was subsequently lowered to 2 μg/mL. NCI-H358 tTS/rtTA cells were then transduced with lentivirus encoding tet-inducible HER2, RET M918T, KRAS^G12C^, or GFP. Cells were cultured in growth medium containing puromycin (2 μg/mL) and blasticidin (2 μg/mL) starting 48 hours after transduction to maintain the selective pressure for both plasmids. Expression of the RTK transgene or GFP was induced by adding doxycycline (0.1–1 μg/mL) for at least 24 hours. KRAS^G12C^ transgene expression was induced by treatment with doxycycline (2 μg/mL) for 48 hours prior to downstream assays. HER2 overexpression NCI-H358 cells were generated as described and authenticated in February 2022. RET overexpression NCI-H358 cells were generated and authenticated in March 2022. Dox-inducible KRAS^G12C^ overexpression NCI-H358 cells were generated in January 2024 and authenticated in October 2025. NCI-H358 GFP-expressing cells were generated in July 2021 and authenticated in May 2023.

#### Compound Formulation

For *in vitro* studies, all tested agents, including elironrasib and daraxonrasib, were resuspended in dimethyl sulfoxide (DMSO) and used at 10 mmol/L stock concentration. For *in vivo* studies, elironrasib, daraxonrasib, and RMC-7977 single agents and elironrasib + daraxonrasib coformulation were prepared in 10/20/10/60 (%v/v/v/v) DMSO/PEG 400/Solutol HS15/water. Sotorasib solution was prepared in 2% (w/v) HPMC E-50/0.5% Tween-80 in 50 mmol/L Sodium Citrate Buffer, pH 4.0. RMC-4998 single agent and RMC-4998 + RMC-7977 coformulation were prepared in 10/20/10/60 (%v/v/v/v) DMSO/PEG 400/Solutol HS15/2% HPMC E-50 (w/v) 50 mmol/L Sodium Citrate Buffer, pH 4.0. 10/20/10/60 (%v/v/v/v) DMSO/PEG 400/Solutol HS15/water was used for vehicle control groups.

### *In Vitro* Assays

#### 3D Cell Proliferation Analysis

Dox-inducible NCI-H358 RTK or GFP overexpression cells cultured in complete cell culture media with the addition of 1 μg/mL doxycycline were seeded in the wells (2,500 cells/well) of a round-bottom ultra-low attachment 96-well plate, centrifuged at 1,000 rpm for 10 minutes to pellet the cells, and incubated for 72 hours to allow for spheroid formation. Cells were exposed to elironrasib and daraxonrasib in the matrix format, prepared in fourfold serial dilutions across five concentrations and threefold serial dilutions across nine concentrations, respectively, including DMSO control (0.2% v/v) for 120 hours using Tecan D300e digital drug dispenser. Cell viability was assessed using 3D CTG reagent (Promega, G9683) according to the manufacturer’s protocols. Luminescence was detected using a SpectraMax M5 Plate Reader (Molecular Devices). % control = lum_treated_/lum_DMSO_ × 100. Data were plotted as a function of log molar (inhibitor).

#### 2D Cell Proliferation Analysis

Dox-inducible NCI-H358 KRAS^G12C^ overexpression cells treated with/without doxycycline (2 μg/mL) were seeded into tissue culture–treated 384-well plates (Corning, flat-bottom, white) at a density of 500 cells per well in 40 μL of complete growth medium. Plates were incubated overnight at 37°C in a humidified incubator with 5% CO_2_ to allow for cell adherence. Cells were exposed to a 1:3 matrix serial dilution of daraxonrasib (top concentration of 100 nmol/L) and elironrasib (top concentration of 10 nmol/L) or to a DMSO control (0.1% v/v) using a Tecan D300e digital dispenser and incubated for 120 hours at 37°C, with treatments performed in triplicate. Cell viability was assessed using the CTG 2.0 luminescent cell viability assay (Promega, G9243) according to the manufacturer’s instructions. Luminescence was measured using a SpectraMax M5 multimode plate reader (Molecular Devices) with an integration time of 250 milliseconds per well. Luminescence signal was subtracted by the mean D0 luminescence for each condition and then normalized to vehicle-treated wells [% vehicle = (lum_treated_/mean(lum_vehicle_) × 100]. Normalized dose–response curves were generated using GraphPad Prism (RRID: SCR_002798), applying a nonlinear regression model with variable slope (four-parameter logistic equation).

#### Western Blot Analysis

Cells were seeded at 0.3 million cells per well of tissue culture–treated six-well plates. After overnight incubation, compounds or DMSO (0.1% v/v) were added and incubated for the indicated time points. Cells were washed twice with ice-cold PBS, lysed with MSD Tris Lysis Buffer (MSD, R60TX-2), scraped, and collected before centrifugation. All lysis buffers were supplemented with protease and phosphatase inhibitors (Thermo Fisher Scientific, 78444). Lysates were centrifuged at 14,000 × *rpm* for 10 minutes at 4°C. The protein concentrations in supernatants were measured using Pierce BCA Protein Assay Kit (Thermo Fisher Scientific, 23225). Then, all the lysates with equal quantities of protein were denatured at 95°C for 10 minutes after adding LDS sample buffer (Thermo Fisher Scientific, NP0008) and reducing agent (Thermo Fisher Scientific, NP0009). Samples were resolved on 4% to 12% Bis-Tris polyacrylamide gels (Thermo Fisher Scientific, WG1403BOX), and Western blot was performed following the standard protocol.

The following primary antibodies were used at 1:1,000 dilution: anti-phospho-p44/42 MAPK (ERK1/2) T202/Y204 (Cell Signaling Technology, 4370; RRID: AB_2315112), anti-p44/42 (ERK1/2; Cell Signaling Technology, 4696; RRID: AB_390780), anti–phospho-p90RSK Thr359 (Cell Signaling Technology, 8753; RRID: AB_2783561), anti-RSK1/2/3 (Cell Signaling Technology, 9355; RRID: AB_659900), anti-vinculin (Cell Signaling Technology, 13901; RRID: AB_2728768), and anti-RAS (Abcam, ab108602; RRID: AB_10891004). The following secondary antibodies were used as appropriate: goat anti-rabbit IR800-conjugated (LI-COR, 926-32211; RRID: AB_621843) and goat anti-mouse IR680-conjugated (LI-COR, 926-68070; RRID: AB_10956588).

#### Calreticulin and HMGB-1 Assays to Measure ICD

CalR externalization was evaluated by flow cytometry. KPAR1.3 and 3LL-ΔNRAS cells were seeded in 96-well plates (Corning, 3603) at a density of 20,000 cells/well in 100 μL complete DMEM. The next day, cells were treated with compounds of interest: daraxonrasib (9 nmol/L), elironrasib (43 nmol/L), doxorubicin (30 μmol/L), MTX (1 μmol/L). Cells were then incubated for 24 hours at 37°C. Following the incubation, cell supernatants were harvested and transferred to a 96-well U-bottom plate. Adherent cells from stimulation plate were treated with 50 μL TrypLE enzyme per well, incubated at 37C for 3 minutes, and combined with the supernatant. Cells were stained with fixable viability dye (Invitrogen, L34975A) and subsequently stained with anti-CalR antibody (Cell Signaling Technology, 64678; RRID: AB_3719576) or isotype control antibody (Cell Signaling Technology, 9078; RRID: AB_2797697) for 1 hour at 4°C. Cells were analyzed with Cytek Aurora. The frequency of CalR+ live cells was quantified.

HMGB-1 release was measured using the Lumit HMGB1 Immunoassay (Promega, W6110). KPAR1.3 and 3LL-ΔNRAS cells were seeded in 96-well plates (Corning, 3603) at a density of 20,000 cells/well in 100 μL complete DMEM and stimulated the next day with compounds of interest. Following 48 hours of stimulation, supernatants were harvested for analysis using the Lumit HMGB1 kit according to the manufacturer’s instructions.

### *In Vivo* Studies

#### Animal Studies Using Xenograft Tumor Models

Studies were conducted at Cancer Research UK (CRUK) Manchester Institute and the following contract research organizations (CRO): GenenDesign, Pharmaron, WuXi AppTec, Champions Oncology, Charles River Laboratories, and XenoSTART. Studies at CRUK Manchester Institute were carried out in accordance with Home Office Regulations (UK) and the UK Coordinating Committee on Cancer Research guidelines and by approved protocols (Home Office Project license P3ED48266/PP9203264). Animal experiments were overseen by the Cancer Research UK Manchester Institute Animal Welfare and Ethical Review Advisory Board. Eight- to 12-week-old female NOD.Cg-*Prkdc*^*scid*^*Il2rg*^*tm1Wjl*^/SzJ (NSG, RRID: IMSR_JAX:005557) mice from Charles River Laboratories were used for *in vivo* studies at CRUK Manchester Institute. Mice were acclimated to the Cancer Research UK Manchester Institute animal facility for a minimum of 1 week prior to any procedures/treatment. They were group housed in pathogen-free individually ventilated cages in a 12-hour light/12-hour dark cycle maintained at uniform temperature and humidity, with unrestricted access to food and water. All procedures (cell implants, dosing, and caliper measurements) were carried out in the morning on a laminar air flow bench and mice placed back in their home cages. All CDX/PDX mouse studies and procedures at CROs related to animal handling, care, and treatment complied with all applicable regulations and guidelines of the institutional Animal Care and Use Committee (IACUC) at each facility with their approvals. Female BALB/c nude, NOD SCID, NMRI nu/nu, and athymic Nude mice 6 to 8 weeks old were used. Animal vendors include Beijing Vital River/VR Laboratory Animal, Co. LTD., Beijing AniKeeper Biotech, Co. LTD., Shanghai Sino–British SIPPR/BK Laboratory Animal, Co. LTD., Envigo RMS LLC, and Charles River Laboratories.

#### Generation of Xenograft Models

NCI-H2122 (5 × 10^6^ cells) and NCI-H2030 (1 × 10^7^ cells) cancer cells in 100 to 200 μL of PBS/media supplemented with Matrigel (1:1; v/v) were inoculated at the right flank of each immunodeficient mouse to generate subcutaneous CDX. Treatments were initiated when the average tumor volume reached 150 to 200 mm^3^ for tumor growth evaluation, 400 to 500 mm^3^ for single-dose PK/PD, and 500 to 600 mm^3^ for repeat-dose PK/PD studies. Human cancer PDX models at CROs were established using fresh tumor specimens obtained from patients with NSCLC who were provided informed written consent in accordance with protocols approved by the Hospital’s Institutional Ethical Committee. Tumor fragments were subcutaneously serial passaged in immunodeficient mice and cryopreserved for future use. Recovered tumor fragments were implanted into the right flanks of immunodeficient mice. When the average tumor volume reached 100 to 300 mm^3^, treatments commenced via daily oral gavage. For the PDX establishment at CRUK Manchester Institute, tumor samples were collected from patients with NSCLC through informed written consent in accordance with the ethically approved Manchester Cancer Research Centre (MCRC) Biobank protocol (18/NW/0092, project _21COLI_01). The MCRC Biobank is licensed by the Human Tissue Authority (license number: 30004) and has been ethically approved as a research tissue bank by the South Manchester Research Ethics Committee (Ref: 22/NW/0237). Biopsies/tumor pieces were surgically implanted subcutaneously into the flank of immunocompromised mice. When the total tumor burden reached 1,000 mm^3^ or there were demonstrable signs of ill health, the mouse was killed. Tumor fragments or freshly dissociated tumor cells were passaged into NSG mice, and the remainder of the tumor was harvested for IHC analysis or DNA or RNA extraction. For the *in vivo* studies at CRUK Manchester Institute, 100,000 viable PDX cells in 100 μL 1:1 HITES:Cultrex were injected subcutaneously into the right flank of female NSG mice. Individual tumor-bearing animals were allocated via stratified randomization into different cohorts for treatments once tumor volumes reached 170 to 250 mm^3^.

Tumor diameter was measured in two dimensions using a digital caliper, and mice in the study were weighed twice per week. The tumor volume in mm^3^ was calculated based on the following formula: volume = [length × (width)^2^]/2.

#### Animal Studies Using Syngeneic Models

Studies were conducted at Revolution Medicines and HD Biosciences. Studies were approved by the IACUC. All studies were conducted in compliance with the facility’s animal welfare body guidelines and animal use protocols. Female (for KPAR1.3) or male (for 3LL-ΔNRAS) C57BL/6 mice (8–16 weeks old; RRID: IMSR_JAX:000664) were purchased from The Jackson Laboratory and were acclimated for 7 days before cell implantation.

#### Generation of Syngeneic Models

Each mouse was inoculated subcutaneously at the right flank with KPAR1.3 (5 × 10^5^ cells) in 0.1 mL of 50% Matrigel 50% serum-free RPMI or 3LL-ΔNRAS (4 × 10^5^ cells) tumor cells in 0.1 mL of 50% Matrigel 50% serum-free DMEM. Treatments were initiated when the average tumor volume reached 250 mm^3^ (KPAR1.3) or 100 mm^3^ (3LL-ΔNRAS) for tumor growth evaluation, 400 to 1,200 mm^3^ for PD studies, 100 to 800 mm^3^ for assessing TME modulation, and 300 to 500 mm^3^ for TCR-seq studies.

#### *In Vivo* Treatment

For the tumor growth evaluation study, tumor-bearing animals were randomized and grouped for treatments (*n* = 3–15 per group). The vehicle, sotorasib, elironrasib, daraxonrasib, RMC-4998, RMC-7977, elironrasib + daraxonrasib, and RMC-4998 + RMC-7977 at indicated doses were administered daily via oral gavage with a 10 mL/kg dosing volume. Animals were terminated as described previously (13). For PKPD studies (*n* = 3 per time point for each dose), TME analysis studies (*n* = 5 for each dose), and TCR-seq study (*n* = 10 for each dose), animals were randomized and grouped for treatments. RAS-targeted inhibitors as single agents or in coformulation were administered orally once or once per day for multiple times at indicated doses. Anti-mouse PD-1 (clone RMP1-14-CP15) or the mouse IgG2a isotype control from Bio X Cell was dosed at 10 mg/kg intraperitoneally biweekly. Blood, plasma, and tumor tissues were harvested at indicated time points after the last dose. Snap-frozen and formalin-fixed samples were collected following the same protocols as described previously (11, 13).

#### Orthotopic Studies

Each mouse was injected with KPAR1.3 (5 × 10^5^ cells) tumor cells in 0.2 mL serum-free RPMI in the tail vein. Mouse weight was monitored biweekly as a measure of tumor growth. Tumor burden was also assessed by regular computed tomography (CT) scanning of the lungs. Each mouse received a 0.1 mL tail vein injection of ExiTron nano 12000, a nanoparticle contrast agent for high-resolution CT angiography, using a 1 mL, 30-gauge needle. Immediately after the tail vein injection, mice were anesthetized by inhalation of isoflurane and scanned using the Quantum GX2 microCT imaging system (PerkinElmer) at a 72 μm isotropic pixel size. Serial lung images were reconstructed, and tumor volumes were subsequently analyzed using Analyze 14.0 (AnalyzeDirect). Randomization was performed when at least two tumor nodules of 1 mm^3^ each were detected by CT scan (24 days after tumor inoculation). Lungs were harvested at 4 hours after 4 days of treatment and fixed in 10% neutral buffered formalin for 24 hours and transferred to 70% ethanol. The lungs were divided into four segments for paraffin embedding: left apical, right and middle, left basal, and right basal lobes.

#### Gold Standard Vaccination-Rechallenge Experiments to Evaluate ICD

KPAR1.3 cells were treated with either elironrasib, daraxonrasib (1 μmol/L, 72 hours), or MTX (5 μmol/L, 48 hours). Treated cells were harvested, both adherent and nonadherent fractions pooled, and washed three times with PBS. Cells were then counted, viability-assessed with ViCell, and injected subcutaneously in the right flank at a density of 5 × 10^5^ cells per mouse in 0.1 mL of serum-free DMEM. Freeze-thawed KPAR1.3 cells were used as a negative control, as freeze-thawing results in cell death without ICD induction. To generate freeze-thawed cells, KPAR1.3 cells were flash-frozen in liquid nitrogen, thawed three times, and then filtered, counted, and inoculated into mice. Seven days following vaccination, mice were subcutaneously inoculated with 1 × 10^6^ KPAR1.3 cells in 0.1 mL of 50% Matrigel 50% serum-free RPMI in the left flank. Tumor measurements were taken twice per week.

#### *In Vivo* Study Data Analysis

In tumor volume plots, the average tumor volume of each group in CDX models was plotted over the course after implantation, whereas the *x*-axis started on the first day of treatment in PDX and syngeneic models. Control and treatment groups were compared by two-way repeated-measures ANOVA (Dunnett’s multiple comparisons test) on the last measurement day of the control group. The percentage change in body weight for each animal on a given day was determined as [(body weight on test article administration end date/body weight on test article administration start date) − 1] × 100. The percentage of the mean tumor volume change from baseline was graphed in the waterfall plots. The mRECIST was applied to call the initial response in each model following 28 ± 2 days of treatment or when control tumors reached two tumor doublings (whichever was later). Tumor response was called based on the percentage of mean tumor volume change from baseline and categorized into four criteria: progressive disease (mPD), stable disease (mSD), partial response (mPR), and complete response (mCR), yielding an ORR [ORR = (mCR + mPR)/total treated] and disease control rate (mCR + mPR + mSD/total treated). The mRECIST score was determined based on % mean tumor volume change, mCR is more than 80% regression, mPR is between 30% and 80% regression, mSD is between 30% growth and 30% regression, and mPD is more than 30% growth (13, 35). Progression is defined as tumor volume doubling from baseline and represented with Kaplan–Meier plots. Log-rank test was used to compare vehicle control with treatment groups.

#### *In Vivo* PD Analysis by DUSP6 qPCR

∼20 mg of tumor tissues was homogenized in a high-throughput tissue grinder (Scientz, Scientz-48), followed by RNA purification using RNeasy Mini Kit (QIAGEN, 74106) and reverse transcription using High-Capacity cDNA Reverse Transcription Kit (ABI, 4368813) according to the manufacturer’s protocols. Subsequent qPCR analysis was performed using TaqMan Gene-Expression Master Mix (ABI, 4369542) and TaqMan primers specific to *DUSP6* (human-Hs00737962_m1, FAM-MGB) and *18S* (*RNA18S1*; human_Hs99999901_s1, FAM-MGB, used as an internal control gene). The qPCR procedure and following data analysis were described in previous publication (13).

#### Digital PCR

Genomic DNA (gDNA) was extracted from at least 20 mg of tumor tissue using QIAamp DNA Mini Kit (QIAGEN, cat. #51304) or DNeasy Blood & Tissue Kit (QIAGEN, cat. #69504). A volume of 10 ng of gDNA was included for digital PCR (dPCR) using the Naica system multiplex digital PCR (Stilla) or Thermo Fisher Scientific QuantStudio Absolute Q Digital PCR system (RRID: SCR_025738) following the manufacturer’s protocol. Probes and primers for the following genes were included in the multiplexed droplet dPCR (ddPCR) of LUN055 end-of-study tumors in Fig. 2E: *KRAS*^*WT*^ [dPCR mutation detection assay KRAS WT for p.G12C, human (APExBIO, AA100902-WT)], *KRAS*^*G12C*^ [dPCR mutation detection assay KRAS mutant for p.G12C, human (APExBIO, AA100902-MU)], and *RPP30* (*RPP30* probe, 5′-Cy5-TTCTGACCTGAAGGCTCTGCGC-3′; *RPP30* primer F, 5′-TCAGCATGGCGGTGTTT-3′; *RPP30* primer R, 5′-TGGCATGAGGTTGGCCA-3′). The results were normalized to *RPP30* for copy-number assessment. For plate-based multiplexed dPCR of 8 baseline *KRAS*^*G12C*^-mutant NSCLC xenograft tumors in Supplementary Fig. S1B, the following kits were used: *KRAS*^*WT*^ and *KRAS*^*G12C*^ (Absolute Q Liquid Biopsy dPCR Assays, Thermo Fisher Scientific, A53732, Assay ID DGH49PW); *RNase P* (Thermo Fisher Scientific, 4485714). The results were normalized to *RNase P* for copy-number assessment.

#### Mass Spectrometry for KRAS^G12C^ Protein Cross-linking

Flash-frozen tumor pieces were collected for profiling KRAS^G12C^ protein target engagement by elironrasib via targeted LC-MS/MS. Samples were resuspended in lysis buffer (100 mmol/L HEPES pH 8, 8 mol/L guanidine, 1x HALT protease inhibitor cocktail) and homogenized via bead beating. Following BCA analysis, an equal amount of total protein was aliquoted from each sample for downstream sample preparation (100 μg total protein). To each 100 μg total protein sample, 10 ng of heavy KRAS^G12C^ recombinant protein was spiked in. Samples were reduced with DTT (5 mmol/L final) and alkylated with iodoacetamide (30 mmol/L final) before being buffer-exchanged into digestion buffer (0.5 mol/L guanidine and 100 mmol/L HEPES pH 8) via gel filtration (Zeba Plates, Thermo Fisher Scientific). Samples were digested with trypsin/LysC (1:50 enzyme:protein), and the digests were desalted on a C18 column (Waters Sep-Pak) prior to LC-MS/MS acquisition. Levels of KRAS peptides were quantified via custom PRM assay via LC-MS/MS (Orbitrap Lumos Eclipse).

#### Mouse Blood, Plasma, and Tissue Sample Bioanalysis

Systemic or tumor homogenate concentrations of elironrasib and daraxonrasib were determined using LC-MS/MS methods. For the plasma analysis of elironrasib, an aliquot of plasma was extracted with a 20× volume of acetonitrile/methanol (1:3; v/v) containing internal standard (IS, 100 ng/mL verapamil or 100 ng/mL tolbutamide). For the whole blood analysis of daraxonrasib, an aliquot of whole blood was diluted with 3 volumes of water and then extracted by protein precipitation with a 20× volume of acetonitrile/methanol (1:3; v/v) containing IS (100 ng/mL verapamil). For elironrasib and daraxonrasib tumor analysis, tumor homogenate was prepared by homogenizing tumor tissues with nine volumes of homogenizing solution (1X PBS, pH = 7.4). An aliquot of tumor homogenate was extracted with a 10× or 20× volume of acetonitrile/methanol (1:3; v/v) containing IS (100 ng/mL verapamil or 100 ng/mL tolbutamide). After thorough mixing and centrifugation, the supernatant from plasma, whole blood, or tumor homogenate samples was directly analyzed on a Sciex 6500 Plus triple quadrupole mass spectrometer equipped with an ACQUITY UPLC system. An ACQUITY UPLC BEH C18 1.7 μm (2.1 × 50 mm) column was used with gradient elution for compound separation. Analytes and IS were detected by positive electrospray ionization using multiple reaction monitoring (elironrasib: m/*z* 1,012.6/633.2 or 506.8/633.3; daraxonrasib: m/*z* 811.4/779.8; verapamil: m/*z* 455.2/164.9; and tolbutamide: 271.10/155.1). The lower limit of quantification was 2 or 5 ng/mL, and the calibration range was 2 to 1,000 ng/mL, 5 to 1,000 ng/mL, or 2 to 3,000 ng/mL in plasma, whole blood, and tumor homogenate, respectively.

#### PK Analysis

PK parameters were calculated by noncompartmental analysis using Phoenix WinNonlin v8.3.5 software (Certara, NJ; RRID: SCR_024504). Concentrations reported as below the quantification limit were treated as missing for PK analysis. Area under the concentration time curve (AUC) values were estimated using the linear trapezoidal method. AUC_0-last_ values were calculated from the dosing time to the last quantifiable concentration. Maximum concentration (C_max_) was recorded as observed.

#### PK/TE/PD Modeling

A mathematical PK/TE/PD model was developed to compare pathway suppression of either single agent or combination treatment of elironrasib and daraxonrasib. The output of the model was the relative *DUSP6* mRNA level, which was used as a representative of the total pathway suppression. The model captured the observed blood or plasma systemic PK, tumor PK, KRAS^G12C^ TE, and human *DUSP6* mRNA expression data from tumor tissues. The model was trained by datasets involving multiple dose levels with single or repeat dosing for both agents in the settings of monotherapy and combination therapy.

A schematic representation of the model is shown in Supplementary Fig. S4A. The model accounted for both single-agent treatment and combination treatment by accounting for individual component PK and a KRAS inhibition model which included both covalent and noncovalent mechanisms simultaneously. The data used to train the model are provided in Supplementary Table S6. A brief summary of the model development was provided; however, the full mathematical description of the model along with the differential equations and the estimated parameters are provided in Supplementary Methods.

For systemic PK modeling, the measured concentrations of elironrasib in the plasma and the measured concentrations of daraxonrasib in the whole blood were fit by two separate two-compartment models with first-order absorption and elimination. For each agent, the following parameters were estimated via naïve pooling of the data: the first-order absorption constant *k*_*a*_, the apparent central and peripheral volumes (*V*_*c*_ and *V*_*p*_), the apparent first-order clearance (*Cl*), and the apparent intercompartmental clearance (*Q*). The model assumed that the systemic PK of either agent was identical upon single-agent or combination administration.

For tumor PK modeling, the measured total concentrations of each agent in tumor homogenate were used for fitting a single-effect compartment model. Each effect compartment had inflow and outflow rates, *k*_*ct*_ and *k*_*tc*_, respectively. For daraxonrasib, the inflow rate was scaled by a nonlinear blood–plasma partitioning function and was dependent on the dose of elironrasib to match observed data. For elironrasib, the inflow rate was dependent on its own dose. For both agents, the inflow rate was adjusted by the respective fraction unbound in the plasma.

For target engagement modeling, the measured percentage of non–cross-linked KRAS^G12C^ remaining compared with controls was used for fitting. The cross-linking activity of elironrasib to KRAS^G12C^ was assumed to be unaffected by the presence of daraxonrasib. Therefore, KRAS^G12C^ TE was represented via a turnover model along with covalent inhibition driven by the tumor PK of elironrasib. The estimated parameters included the resynthesis rate of KRAS^G12C^*k*_*in*_, the inactivation rate *k*_*inact*_, and the apparent dissociation ratio *K*_*I*_.

For *DUSP6* PD modeling, the measured percentage of *DUSP6* mRNA expression levels compared with controls was used for fitting. In the model, the remaining non–cross-linked KRAS^G12C^ was available to be noncovalently bound by elironrasib or daraxonrasib. It was assumed that *DUSP6* mRNA levels were directly proportional to the remaining amount of the non–cross-linked and unbound species. The inhibitory effects of elironrasib and daraxonrasib were parameterized via the apparent dissociation constants *K*_*I*_ and *K*_*d*_, respectively.

We utilized a sequential fitting approach in which systemic PK was fitted for each agent individually, and it was used as an input for fitting the tumor PK of each agent. Finally, the tumor PK was used to drive KRAS^G12C^ TE and *DUSP6* PD which were fitted simultaneously by naïve pooling of the TE/PD data. *KRAS*^*G12C*^ amplification was modeled by increasing the resynthesis rate *k*_*in*_. The output represented by *DUSP6* mRNA levels was compared against the unamplified untreated baseline.

#### IHC

Tumor fragments were harvested from mice after indicated treatments, fixed in 10% neutral buffered formalin at room temperature for up to 24 hours, stored at 70% ethanol, and then processed into paraffin blocks. Tissue sections (4 μm) were stained on the Bond III (Leica Biosystems) or Biocare intelliPATH automated staining platform using the manufacturer’s recommended settings. The primary antibodies used in IHC staining are listed in Supplementary Table S7. NCI-H2122 tissues sections were incubated with antibody diluent/blocking solution (WiSee Biotechnology, cat. #A10015-100) to block nonspecific background. BOND IHC Polymer Detection Kit (Leica Biosystems, cat. #DS9800) was used to detect rabbit primary antibodies. DAB-stained slides were scanned and digitized with KF-PRO-020 whole-slide scanner at 200× magnification. 3LL-ΔNRAS tissue sections were stained using the Leica Bond RX automated stainer (Leica Microsystems), following a standard operating procedure and a fully automated workflow. Endogenous peroxidase activity was blocked using a peroxide block buffer (Leica Microsystems). Detection was carried out using DAB rabbit secondary reagents—polymer, DAB refine, and hematoxylin (Leica, DS9800)—according to the manufacturer’s protocol. The slides were visualized using a GT450 slide scanner (Leica Microsystems). All the other tissues sections were incubated with Biocare Peroxidase Blocker (Biocare, cat. #PX968) and Background Punisher (Biocare, cat. #BP974) to block nonspecific background. MACH4 HRP-Polymer Detection System (Biocare, cat. #M4U534 or MRH534) was used to detect rabbit primary antibodies. DAB-stained slides were scanned and digitized with a Huron TissueScope LE120 whole-slide scanner at 200× magnification.

#### Whole-Slide Image Analysis

The EpCAM/pan-cytokeratin(panCK) staining was used to identify epithelial cells. First, the area to be analyzed was delineated, excluding necrotic regions. The random forest Tissue Classifier from the HALO Image Analysis package was used to identify the tumor compartment. Three classes were created: glass, tumor (EpCAM-positive/panCK-positive), and stroma. The tumor class mark was then copied onto the serial sections stained with various markers to perform the image analysis only on the tumor compartment.

Quantification of Ki67 was performed with HALO Image Analysis software from Indica Labs using the Cytonuclear module. The analysis was performed only on the tumor compartment copied from the EpCAM slide. The software was tuned to detect all the nuclei based on the hematoxylin stain (blue color) and detect positive DAB staining (brown color). Total positivity was plotted and subjected to statistical analysis using GraphPad Prism (Dunnett multiple comparisons test; RRID: SCR_002798).

Quantification of p-ERK, p-S6, and CC3 was performed with HALO Image Analysis software from Indica labs using the Area Quantification module. The analysis was performed only on the tumor compartment copied from the EpCAM slide. The software was tuned to detect positive DAB staining (brown color). The percentage of area positivity was chosen to represent the results (area positive for brown/total area) and subjected to statistical analysis using GraphPad Prism (Dunnett multiple comparisons test).

Quantification of CD8, CD4, and granzyme B was carried out using Halo v4.0.5107 software from Indica Labs. Positive and negative cells were detected using the Halo Multiplex IHC algorithm v3.4.9 by first defining the settings for the hematoxylin counterstain, followed by setting thresholds to detect the CD4 stain positivity (Halo threshold settings 0.135), CD8 stain positivity (Halo threshold settings 0.16), and granzyme B positivity (Halo threshold settings 0.3135).

#### DNA FISH

Formalin-fixed, paraffin-embedded (FFPE) sections (5 μm) were dewaxed and treated using a FISH pretreatment kit (Abbott Laboratories, 02J06-030) to prepare target tumor DNA for hybridization. Briefly, sections were incubated with acid and heat before digestion with protease and then dehydrated and dried. Dual-labeled human *KRAS*/chromosome 12 centromeric probes from Empire Genomics were mixed with hybridization buffer and applied to FFPE sections. Slides were denatured at 78°C for 6 minutes, hybridized overnight at 37°C and then washed with 0.4× SSC (saline sodium citrate) at 73°C for 2 minutes, followed by another washing with 2× SSC at room temperature for 2 minutes. Tissues were countered stained with DAPI and imaged at 63×.

#### RNA ISH

*KRAS* RNA ISH was performed on 5 μm FFPE tumor sections using HCR RNA ISH probes from Molecular Instruments on a Biocare Oncore Pro X stainer. Dewaxed slides were incubated in Tris-EDTA buffer at 95°C for 15 minutes and then prehybridized with hybridization buffer for 10 minutes. Probes (0.4 pmol) were mixed with hybridization buffer and incubated overnight at 37°C. Slides were washed with a series of 5× sodium chloride sodium citrate buffer with 0.1% Tween-20 (5× SSCT) and then signal was amplified by hybridizing fluorescently labeled hairpins overnight at room temperature. Slides were again washed with 5× SCCT and imaged at 40×.

#### Flow Cytometry Analysis

Tumors were collected at 24 hours after 8 days of indicated treatment. Fresh tumors were minced, processed with the Dri Tumor & Tissue Dissociation Reagent from BD Biosciences, and homogenized with the gentleMACS Dissociator. Tumor cell suspensions were incubated at 4°C for 30 minutes with Mouse BD Fc Block (Clone 2.4G2 from BD Pharmingen), 10 minutes with Blue Dead Cell Stain Kit (Invitrogen), and 30 minutes in cell staining buffer. Antibodies used are listed in Supplementary Table S8. Cells were analyzed on a 4-laser Cytek Aurora (Cytek Biosciences; RRID: SCR_019826), and data analysis was done using SpectroFlo (Cytek Biosciences; RRID: SCR_025494) and FlowJo (FlowJo LLC.; RRID: SCR_008520).

#### TCR Variable β Chain Sequencing

Sequencing of the CDR3 regions of human TCRβ chains was performed using Adaptive Immunosequencing (Adaptive Biotechnologies). Extracted gDNA was amplified in a bias-controlled multiplex PCR, followed by high-throughput sequencing. Sequences were collapsed and filtered to identify and quantitate the absolute abundance of each unique TCRβ CDR3 region for further analysis as described previously (70).

#### Statistical Analyses of TCRβ Sequencing Results

Two quantitative components of diversity were compared across samples in this study. First, Simpson clonality was calculated on productive rearrangements by

$$\sqrt{\overset{R}{\underset{i\;=\;1}{\sum }}p_{i}^{2}}$$

In which R is the total number of productive rearrangements and $p_{i}$ is the productive frequency of rearrangement i. Values of Simpson clonality range from 0 to 1 and measure how evenly receptor sequences (rearrangements) are distributed. Clonality values approaching 0 indicate an even distribution of frequencies, whereas values approaching 1 indicate an increasingly asymmetric distribution in which one to a few clones are present at high frequencies. Second, sample richness was calculated as the number of unique productive rearrangements in a sample after computationally downsampling to a common number of templates to control for variation in sample depth or T-cell fraction. Repertoires were randomly sampled without replacement five times, and the mean of these values is reported as downsampled rearrangements. T-cell fraction was calculated by taking the total number of T-cell templates detected and dividing by the total number of nucleated cells. The total number of nucleated cells is determined using a panel of reference genes as part of immunosequencing. Clonal expansion/contraction was calculated according to a binomial distribution framework as described previously (71). In brief, for each unique rearrangement, we treat the combined template count between the two samples being compared as a fixed number of “trials” in a two-sided binomial test. We calculate the probability of the observed template counts in each sample under the null hypothesis that these templates are evenly distributed between the two samples relative to their respective repertoire sizes (i.e., total productive templates). Clones that are more unequally distributed relative to this expected proportion will result in a lower binomial probability. The Benjamini–Hochberg procedure was used to control the false discovery rate (FDR) at 0.01. All statistical analyses were performed in R version 4.1.

#### Bioinformatics Analyses

Whole-exome sequencing and bulk RNA-seq (IQVIA, Novogene) analyses were performed to ascertain gene mutations and gene expression, respectively. DNA mutation calling was accomplished with TNSeq using the hg38 version of the human genome. For CDX models, gene mutation and gene expression data were obtained from the 23Q4 release of the DepMap Data Portal.

Transcript-level quantification was performed using Salmon (version 1.0.8) in quasi-mapping mode (72). Reference transcriptomes used for quantification were derived from the mouse genome (GRCm38) or from the human genome (hg38) depending on the model. The transcript-level counts generated by Salmon were imported into R, and genes with low expression across the samples were filtered out before downstream analysis.

Normalization of gene-level counts was performed using the trimmed mean of M-values method, after which log-transformed counts per million (CPM) values were calculated using the CPM function from the edgeR package (RRID: SCR_012802; ref. 73). These values were used to calculate *z*-score normalized pathway activity scores for the MAPK signaling pathway derived from the PROGENy database (version 1.26.0; ref. 74). PROGENy pathways were calculated with the appropriate species flag—either human or mouse—for the different models.

Similarly, gene set variation analysis (GSVA) was performed using the GSVA package (version 1.52.3; ref. 75) with log-CPM values to compute cell-cycle scores derived from the Seurat package (RRID: SCR_016341; ref. 76). Heatmaps were generated using the ComplexHeatmap package (RRID: SCR_017270) in R (77, 78).

## Supplementary Material

Supplementary Methods 1 Detailed PK-TE-PD modeling method

Table S1 KM in xenograft models

Table S2 TV and BW in xenograft models

Table S3 IHC staining quantification in xenograft tumors

Table S4 PK in NCI-H2122 xenograft tumor-bearing mice

Table S5 TE and human DUSP6 mRNA expression in xenograft tumors

Table S6 Raw data used for PK_TE_PD modeling

Table S7 Primary antibody list for IHC staining

Table S8 Antibodies for flow cytometry

Supplementary Figures S1-S7 Supplementary Figure 1. The RAS(ON) inhibitor doublet induces deep and durable responses in KRASG12C-mutant NSCLC models and is tolerated based on animal body weight assessment. Supplementary Figure 2. The RAS(ON) inhibitor doublet demonstrates combinatorial benefit in vitro. Supplementary Figure 3. The RAS(ON) inhibitor doublet demonstrates combinatorial benefit in vivo. Supplementary Figure 4. Graphical representation of the combined PK/TE/PD model. Supplementary Figure 5. Elironrasib and daraxonrasib synergize and drive ICD and immune dependent CR in the KPAR1.3 model. Supplementary Figure 6. The RAS(ON) doublet increases depth of response in the 3LL-ΔNRAS model. Supplementary Figure 7. Immuno-sequencing (TCRB assay) of gDNA extracted from 3LL-ΔNRAS tumors collected at 24 hours post 8 days of daily oral treatment with vehicle, elironrasib at 30 mg/kg or daraxonrasib at 25 mg/kg or the RAS(ON) doublet.

## Data Availability

RNA-seq data for NCI-H2122 subcutaneous xenograft tumors and 3LL-ΔNRAS subcutaneous syngeneic tumors treated with RAS(ON) inhibitors are available under the Gene Expression Omnibus database GSE315010. TCR sequencing data for 3LL-ΔNRAS subcutaneous syngeneic tumors treated with RAS(ON) inhibitors are available at immuneACCESS https://doi.org/10.21417/CB2026CD. Data used to generate analyses and visualization in this publication are available within the article and its supplementary data files. Revolution Medicines will not provide access to patient-level data if there is a reasonable likelihood that individual patients could be reidentified.
