# Supplementary material for: Abrogation of Oncogenic RAS Signaling by a RAS(ON) Inhibitor Doublet Primes Immune-Refractory KRASG12C-Mutant NSCLC for Immune Checkpoint Blockade
Source: Cancer Discov. 2026 Feb 11;16(6):1152–75. doi: 10.1158/2159-8290.CD-25-1616 (PMC13223541; doi:10.1158/2159-8290.CD-25-1616)
Supplement: Supplementary Methods 1 — Detailed PK-TE-PD modeling method [file cd-25-1616_supplementary_methods_1_suppsm1.pdf]

SUPPLEMENTARY METHODS

We provide here the technical details of the PK/TE/PD mathematical model presented in the main text. This supplementary note is organized as follows: we first provide the mathematical description of the model, then we list the parameter estimates, and finally we describe the data set used to fit the model.

1. Mathematical description of the model

The model is depicted schematically in suppelementary Figure 4. The model incorporates several datasets using a sequential fitting approach as follows:

1. Systemic PK: Two independent compartmental PK models were fitted against the measured whole blood and plasma PK for daraxonrasib and elironrasib, respectively. This corresponds to Eqs. (1)-(3) and Eqs. (4)-(6), respectively.
2. Tumor PK: Two effect compartment models for each agent were fitted against their respective measured tumor PK. This corresponds to Eq. (7) and Eq. (8), respectively.
3. TE and PD data: A combined TE/PD model was developed and fitted against the TE and *DUSP6* mRNA data. This corresponds to Eqs. (9)-(10).

We describe the details of each submodel next.

1.1 Systemic PK models

Daraxonrasib. The whole blood PK of daraxonrasib is represented by a two-compartmental model with first order absorption and clearance as follows:

$$\frac{dA_d}{dt} = -k_{a,d}A_d, \tag{1}$$

$$V_{c,d}\frac{dC_{c,d}}{dt} = k_{a,d}A_d - (C\ell_d)C_{c,d} - Q_d(C_{c,d} - C_{p,d}), \tag{2}$$

$$V_{p,d}\frac{dC_{p,d}}{dt} = Q_d(C_{c,d} - C_{p,d}), \tag{3}$$

where the parameters are defined as follows

- A_d (nmole) is the amount of daraxonrasib at the absorption site. Daraxonrasib's dose in nanomoles ($d_{nmol,d}$) is calculated as follows:

  $$d_{nmol,d} = 10^6 \frac{d_{mg/kg,d}}{\mathrm{mw}_d} w_{\mathrm{mouse}},$$

  where $d_{mg/kg,d}$ is daraxonrasib's dose in mg/kg, mw_d is daraxonrasib's molecular weight fixed at 811.066 g/mol, and w_{mouse} is the nominal mouse weight fixed at 0.025 kg.
- $C_{c,d}$ (nM) is the concentration of daraxonrasib in the central compartment which is initialized to zero at time=0. It is fitted against the whole blood PK of daraxonrasib.

- $C_{p,d}$ (nM) is the concentration of daraxonrasib in the peripheral compartment which is initialized to zero at time=0.
- $V_{c,d}$ (L) is the volume of the central compartment in daraxonrasib's whole blood PK model.
- $V_{p,d}$ (L) is the volume of the peripheral compartment in daraxonrasib whole blood PK model.
- $k_{a,d}$ (1/h) is daraxonrasib's absorption rate constant.
- $\mathcal{C}\ell_d$ (L/h) is daraxonrasib's clearance in the central compartment.
- Q_d (L/h) is daraxonrasib's inter-compartmental clearance.

The parameter estimates are listed in Table SM1.

Elironrasib. The Plasma PK of elironrasib is represented by a two-compartmental model with first order absorption and clearance as follows:

$$\frac{dA_e}{dt} = -k_{a,e}A_e \tag{4}$$

$$V_{c,e}\frac{dC_{c,e}}{dt} = k_{a,e}A_e - (\mathcal{C}\ell_e)C_{c,e} - Q_e(C_{c,e} - C_{p,e}) \tag{5}$$

$$V_{p,e}\frac{dC_{p,e}}{dt} = Q_e(C_{c,e} - C_{p,e}), \tag{6}$$

where the parameters are defined as follows

- A_e (nmole) is the amount of elironrasib at the absorption site. Elironrasib's dose in nanomoles ($d_{nmol,e}$) is calculated as follows:

  $$d_{nmol,e} = 10^6 \frac{d_{mg/kg,e}}{\text{mw}_e} w_{\text{mouse}},$$

  where $d_{mg/kg,e}$ is elironrasib's dose in mg/kg, and mw_e is elironrasib's molecular weight fixed at 1012.3 g/mol.
- $C_{c,e}$ (nM) is the concentration of elironrasib in the central compartment which is initialized to zero at time=0. It is fitted against the plasma PK of elironrasib.
- $C_{p,e}$ (nM) is the concentration of elironrasib in the peripheral compartment which is initialized to zero at time=0.
- $V_{c,e}$ (L) is the volume of the central compartment in elironrasib's plasma PK model.
- $V_{p,e}$ (L) is the volume of the peripheral compartment in elironrasib plasma PK model.
- $k_{a,e}$ (1/h) is elironrasib's absorption rate constant.
- $\mathcal{C}\ell_e$ (L/h) is elironrasib's clearance in the central compartment.
- Q_e (L/h) is elironrasib's inter-compartmental clearance.

The parameter estimates are listed in Table SM1.

1.2 Tumor PK models

Daraxonrasib. The tumor PK of daraxonrasib is represented by a single effect compartment model with first order absorption and clearance as follows:

$$\frac{dC_{t,d}}{dt} = k_{ct,d}\left(1 + \frac{\alpha_1 d_{mg/kg,e}}{d_{mg/kg,e} + \alpha_2}\right)\frac{f_{up,d}}{\text{BPP}}C_{c,d} - k_{ct,d}C_{t,d} \tag{7}$$

where the parameters are defined as follows

- $C_{t,d}$ (nM) is the concentration of daraxonrasib in the tumor tissue which is initialized to zero at time=0
- $k_{ct,d}$ (1/h) is the inflow rate from the unbound plasma to the tumor in the absence of elironrasib.
- $k_{ct,d}$ (1/h) is the outflow rate from the tumor.
- $f_{up,d}$ (dimensionless) is fraction unbound in the plasma which is measured to be 0.011.
- BPP (dimensionless) is the blood-to-plasma partitioning ratio which is estimated as follows

$$\text{BPP} = 5.5 - 5.2\frac{C_{c,d}}{1819 + C_{c,d}}.$$

- α_1, α_2 (dimensionless, mg/kg) are empirical constants to account for daraxonrasib's tumor PK dependence on elironrasib's dose.

The parameter estimates are listed in Table SM1.

Elironrasib. The tumor PK of elironrasib is represented by a single effect compartment model with first order absorption and clearance as follows:

$$\frac{dC_{t,e}}{dt} = k_{ct,e}\left(1 + \alpha_e d_{mg/kg,e}\right) f_{up,e}C_{c,e} - k_{ct,e}C_{t,e} \tag{8}$$

where the parameters are defined as follows

- $C_{t,e}$ (nM) is the concentration of daraxonrasib in the tumor tissue which is initialized to zero at time=0
- $k_{ct,e}$ (1/h) is the inflow rate from the unbound plasma to the tumor.
- $k_{ct,e}$ (1/h) is the outflow rate from the tumor.
- $f_{up,e}$ (dimensionless) is fraction unbound in the plasma which is measured to be 0.02.
- α (1/(mg/kg)) is an empirical constant to account for the observed dependence of elironrasib's tumor PK on its own dose.

The parameter estimates are listed in Table SM1.

1.3 A semi-mechanistic TE/PD model

As shown in supplementary Figure 4, the TE/PD model accounts for both agents simultaneously.

Mechanistically, each agent forms a binary complex with CypA, which in turn forms a tricomplex with KRAS G12C. Since CypA is highly abundant in the cellular environment, *we assume that the free concentration of the binary complex of each agent is linearly proportional to the corresponding total tumor concentration.*

In addition, we assume that KRAS G12C resynthesis follows a simple turnover model with zeroth-order synthesis and first-order degredation. In the presence of the agents, KRAS G12C has four different species shown in supplementary Figure 4: free, non-covalently-bound to elironrasib, non-covalently-bound to daraxonrasib, and crosslinked to elironrasib. Note the first three species are in continuous equilibrium with each other forming the pool of non-crosslined KRAS G12C, while the last species is formed irreversibly and does not return to the aforementioned pool.

We assume that elironrasib's binary complex follows a standard two-step crosslinking process with KRAS G12C. It forms a transient non-covalent bond with a dissociation constant K_I and then it crosslinks irreversibly with inactivtation rate k_{inact}. In addition, we assume that elironrasib's crosslinking process is independent of the presence of daraxonrasib. Hence, by setting the fast non-covalent binding/unbinding events to a quasi-steady-state, we get the standard covalent binding model with turnover:

$$\frac{dR}{dt} = k_{in} - k_{out}R - k_{inact}R\frac{C_{t,e}}{C_{t,e} + K_I}, \tag{9}$$

where the parameters are defined as follows

- R (dimensionless) is level of non-crosslinked KRAS G12C relative to baseline. It is fitted to the measured TE data. In the model, it accounts for the three non-crosslinked species as shown in supplementary Figure 4.
- k_{in} (1/h) is the resynthesis rate of KRAS G12C.
- k_{out} (1/h) is the degradation rate of KRAS G12C. Since we assume a 100% baseline for KRAS G12C, k_{out} is normalized as $k_{out} = k_{in}/100$.
- k_{inact} (1/h) is the irreversible crosslinking rate of elironrasib.
- K_I is the *apparent* dissociation constant governing the binding of elironrasib's binary complex to KRAS G12C.

We assume that *DUSP6* mRNA is proportional to free KRAS G12C. Accounting for non-covalent binding with elironrasib and daraxonrasib, free KRAS G12C can be written as

$$R_{free} = \frac{R}{1 + \frac{C_{t,d}}{K_d} + \frac{C_{t,e}}{K_I}}, \tag{10}$$

where

- R_{free} (dimensionless) is level of free KRAS G12C relative to baseline. It is fitted to the measured *DUSP6* data.
- K_d (nM) is the *apparent* dissociation constant governing the binding of daraxonrasib's binary complex to KRAS G12C.

Equations (9)-(10) are fitted simultaenousely to the TE and *DUSP6* data, respectively.

2. Parameter fits

The overall model is represented by Eqs. (1)-(10). The fitted parameters are listed in Table SM1. All the fitting and simulations were run using MATLAB/Simbiology V2024b. For sysmetic and tumor PK fits, a log-additive error function was used, while for TE/PD fits an additive error function was used. When choosing the fitting algorithm, the one that resulted in the lowest Akaike information criterion (AIC) was used. For evaluating the accuracy in estimating the target inhibition, we employ the Mean Absolute Error (MAE) which is the average absolute difference between the observed and predicted data. We calculated the MAE for the TE/PD data to be 0.033 across the dosing interval. This means that our predictions are, on average, within 3.3% of the observed data.

Parameter symbol	unit	estimate±standard error
$V_{c,d}$	L	0.1286±0.016
$V_{p,d}$	L	0.0698±0.0092
$\mathcal{C}\ell_d$	L/h	0.0561±0.0003
Q_d	L/h	0.0054±0.0007
$k_{a,d}$	1/h	0.4991±0.0697
$V_{c,e}$	L	0.005±0.00003
$V_{p,e}$	L	0.5865±0.0142
$\mathcal{C}\ell_e$	L/h	0.1057±0.0006
Q_e	L/h	0.0124±0.0012
$k_{a,e}$	1/h	0.384±0.002
$k_{ct,d}$	1/h	321.57±21.3058
$k_{tc,d}$	1/h	0.0708±0.0026
$\alpha_{1,d}$	dimensionless	2.8126±2.9738
$\alpha_{1,d}$	mg/kg	200.0597±286.34
$k_{ct,e}$	1/h	3.0224±0.3566
$k_{tc,e}$	1/h	0.6174±0.0592
α_e	1/(mg/kg)	0.0395±0.0136
k_{in}	1/h	7.05±0.34
K_I	nM	164.99±10.337
K_d	nM	57.13±5.5322
k_{incat}	1/h	1.8798±0.0314

Table SM1: Fitted model parameters.

3. Description of the dataset

The data used to train the model is summarized in Table SM2, while the raw data is provided in supplemntary Table S7.

**Elironrasib (mg/kg)**	**Daraxonrasib (mg/kg)**	**#Dosing Days**	**Systemic PK Samples (h)**	**Tumor PK Samples (h)**	***DUSP6* Samples (h)**	**TE Samples (h)**	**Study#**
200	0	1	0.5,1,4,8,16,24,48	N/A	N/A	N/A	12
200	0	1	0.5,1,4,8,16,24,48	N/A	1, 8,24,48	N/A	13
200	0	7	0.5,1,4,8,16,24,48	N/A	1,8,24,48	N/A	13
0	25	1	0.5,1,4,8,16,24,48	N/A	1,8,24,48	N/A	13
0	25	7	0.5,1,4,8,16,24,48	N/A	1,8,24,48	N/A	13
200	25	1	0.5,1,4,8,16,24,48	N/A	1,8,24,48	N/A	13
200	25	7	0.5,1,4,8,16,24,48	N/A	1,8,24,48	N/A	13
0	3	1	0.5,1,4,8,16,24,48	N/A	1,8,24,48	N/A	14
0	10	1	0.5,1,4,8,16,24,48	N/A	1,8,24,48	N/A	14
25	0	1	0.5,1,4,8,16,24,48	N/A	1,8,24,48	N/A	14
100	0	1	0.5,1,4,8,16,24,48	N/A	1,8,24,48	N/A	14
25	3	1	0.5,1,4,8,16,24,48	N/A	1,8,24,48	N/A	14
25	10	1	0.5,1,4,8,16,24,48	N/A	1,8,24,48	N/A	14
25	25	1	0.5,1,4,8,16,24,48	N/A	1,8,24,48	N/A	14
100	3	1	0.5,1,4,8,16,24,48	N/A	1,8,24,48	N/A	14
100	10	1	0.5,1,4,8,16,24,48	N/A	1,8,24,48	N/A	14
100	25	1	0.5,1,4,8,16,24,48	N/A	1,8,24,48	N/A	14
30	0	1	0.5,1,2,4,8,24,48	2,4,8,24,48	2,4,8,24,48	2,4,8,24,48	20
0	25	1	0.5,1,2,4,8,24,48	2,4,8,24,48	2,4,8,24,48	N/A	20
30	25	1	0.5,1,2,4,8,24,48	2,4,8,24,48	2,4,8,24,48	2,4,8,24,48	20
30	25	7	0.5,1,2,4,8,24,48	2,4,8,24,48	2,4,8,24,48	2,4,8,24,48	20
0	10	1	0.5,1,2,4,8,24,48	2,4,8,24,48	2,4,8,24,48	N/A	21
30	25	1	0.5,1,2,4,8,24,48	2,4,8,24,48	2,4,8,24,48	N/A	21
30	25	7	0.5,1,2,4,8,24,48	2,4,8,24,48	2,4,8,24,48	N/A	21
30	0	1	0.5,1,2,4,8,24,48	4,8,24	4,8,24	4,8,24	23
100	0	1	0.5,1,2,4,8,24,48	4,8,24	4,8,24	4,8,24	23
100	0	7	0.5,1,2,4,8,24,48	2,4,8,24,48	2,4,8,24,48	2,4,8,24,48	23
100	0	17	0.5,1,2,4,8,24,48	2,4,8,24,48	2,4,8,24,48	2,4,8,24,48	23
0	25	1	0.5,1,2,4,8,24,48	4,8,24	4,8,24	N/A	23
0	25	7	0.5,1,2,4,8,24,48	2,4,8,24,48	2,4,8,24,48	N/A	23
0	25	17	0.5,1,2,4,8,24,48	2,4,8,24,48	2,4,8,24,48	N/A	23
30	25	1	0.5,1,2,4,8,24,48	4,8,24	4,8,24	4,8,24	23
100	25	1	0.5,1,2,4,8,24,48	4,8,24	4,8,24	4,8,24	23
100	25	7	0.5,1,2,4,8,24,48	2,4,8,24,48	2,4,8,24,48	2,4,8,24,48	23
100	25	17	0.5,1,2,4,8,24,48	2,4,8,24,48	2,4,8,24,48	2,4,8,24,48	23

Table SM2: Summary of the data sets used to fit the model parameters. The listed sampling times are after the last adiministered dose.
