# Supplementary material for: Abrogation of Oncogenic RAS Signaling by a RAS(ON) Inhibitor Doublet Primes Immune-Refractory KRASG12C-Mutant NSCLC for Immune Checkpoint Blockade
Source: Cancer Discov. 2026 Feb 11;16(6):1152–75. doi: 10.1158/2159-8290.CD-25-1616 (PMC13223541; doi:10.1158/2159-8290.CD-25-1616)
Supplement: Supplementary Figures S1-S7 — Supplementary Figure 1. The RAS(ON) inhibitor doublet induces deep and durable responses in KRASG12C-mutant NSCLC models and is tolerated based on animal body weight assessment. Supplementary Figure 2. The RAS(ON) inhibitor doublet demonstrates combinatorial benefit in vitro. Supplementary Figure 3. The RAS(ON) inhibitor doublet demonstrates combinatorial benefit in vivo. Supplementary Figure 4. Graphical representation of the combined PK/TE/PD model. Supplementary Figure 5. Elironrasib and daraxonrasib synergize and drive ICD and immune dependent CR in the KPAR1.3 model. Supplementary Figure 6. The RAS(ON) doublet increases depth of response in the 3LL-ΔNRAS model. Supplementary Figure 7. Immuno-sequencing (TCRB assay) of gDNA extracted from 3LL-ΔNRAS tumors collected at 24 hours post 8 days of daily oral treatment with vehicle, elironrasib at 30 mg/kg or daraxonrasib at 25 mg/kg or the RAS(ON) doublet. [file cd-25-1616_supplementary_figures_s1-s7_suppsf1.pdf]

Supplementary Figure 1

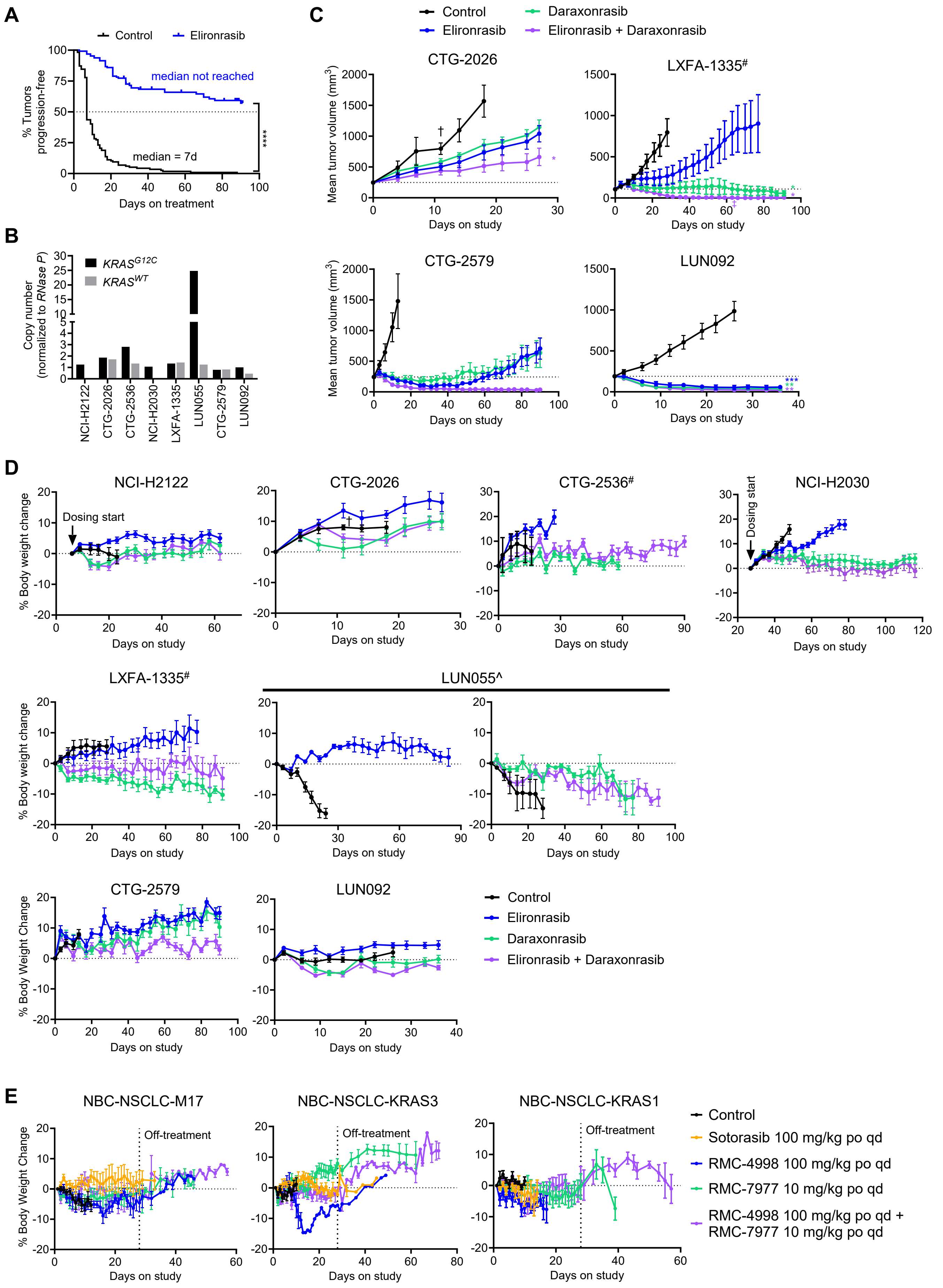

Supplementary Figure 1. The RAS(ON) inhibitor doublet induces deep and durable responses in $KRAS^{G12C}$-mutant NSCLC models and is tolerated based on animal body weight assessment. **A,** Kaplan-Meier analysis of time to tumor doubling on treatment in individual tumor-bearing animals from 26 $KRAS^{G12C}$-mutant NSCLC subcutaneous xenograft models upon daily treatment of elironrasib at 100 or 200 mg/kg for up to 90 days (n = 117 animals in control, n = 128 in elironrasib group). Time to event was determined by the time on treatment until tumor volume doubling from baseline on the survival plot by Kaplan-Meier analysis. Log-rank test was used to compare vehicle control with treatment group (****, $P < 0.0001$). **B,** Relative copy numbers of $KRAS^{WT}$ or $KRAS^{G12C}$ in 8 $KRAS^{G12C}$-mutant NSCLC subcutaneous xenograft tumors (n = 1 per model) at baseline. The relative gene copy numbers were determined by digital PCR and normalized to *RNase P*. **C,** Antitumor activity of elironrasib, daraxonrasib and the combination of elironrasib with daraxonrasib in 4 $KRAS^{G12C}$-mutant NSCLC subcutaneous PDX models including CTG-2026 (n = 6 per group), LXFA-1335 (n = 5 per group), CTG-2579 (n = 6 per group) and LUN092 (n = 6 per group). Tumor-bearing mice were treated with vehicle or RAS(ON) inhibitors (elironrasib at 100 or 200 mg/kg po qd, daraxonrasib at 25 mg/kg po qd and the combination of elironrasib at 100 or 200 mg/kg po qd plus daraxonrasib at 25 mg/kg po qd) for up to 90 days. Mean tumor volumes of each group were plotted over the course of treatment. Vehicle control and specific treatment group were compared by two-way repeated-measures ANOVA on the last measurement day of the vehicle group (*, $P < 0.05$; **, $P < 0.01$; ***, $P < 0.001$). The dotted line indicates the initial average tumor volume. Error bars, SEM. # indicates the model in which elironrasib was dosed at 200 mg/kg. † indicates that 1 animal was terminated upon reaching the tumor burden endpoint. ‡ indicates that 1 animal was terminated due to body weight loss >20% compared to the baseline on day 0 of treatment. **D,** % Body weight change from baseline in 8 $KRAS^{G12C}$-mutant NSCLC subcutaneous xenograft models including NCI-H2122 (n = 8-15 per group), CTG-2026 (n = 6 per group), CTG-2536 (n = 4-6 per group), NCI-H2030 (n = 8 per group), LXFA-1335 (n = 5 per group), LUN055 (n = 3 per group), CTG-2579 (n = 6 per group) and LUN092 (n = 6 per group). Tumor-bearing mice were treated with vehicle or RAS(ON) inhibitors (elironrasib at 100 or 200 mg/kg po qd, daraxonrasib at 25 mg/kg po qd and the combination of elironrasib at 30, 100 or 200 mg/kg po qd plus daraxonrasib at 25 mg/kg po qd) for up to 90 days. The dotted line indicates the initial average % body weight change of each group. Error bars indicate SEM. ^ indicates that 30 mg/kg elironrasib was dosed in the elironrasib + daraxonrasib combination group of LUN055 PDX model. # indicates models in which elironrasib was dosed at 200 mg/kg in both single-agent and combination groups. † indicates that 1 animal was terminated upon reaching the tumor burden endpoint. ‡ indicates 1 animal was terminated due to body weight loss >20% compared to the baseline on day 0 of treatment. **E,** % Body weight change from baseline in 3 $KRAS^{G12C}$-mutant NSCLC subcutaneous PDX models including NBC-NSCLC-M17 (n = 5-7 per group), NBC-NSCLC-KRAS3 (n = 5-6 per group) and NBC-NSCLC-KRAS1 (n = 4-7 per group). Tumor-bearing mice were treated with vehicle or inhibitors (sotorasib at 100 mg/kg po qd, RMC-4998 at 100 mg/kg po qd, RMC-7977 at 10 mg/kg po qd and the combination of RMC-4998 at 100 mg/kg po qd plus RMC-7977 at 10 mg/kg po qd) for up to 28 days followed by off-treatment measurements. The horizontal dotted line indicates the initial average % body weight change of each group. The vertical dotted line indicates treatment stop. Error bars indicate SEM.

Supplementary Figure 2

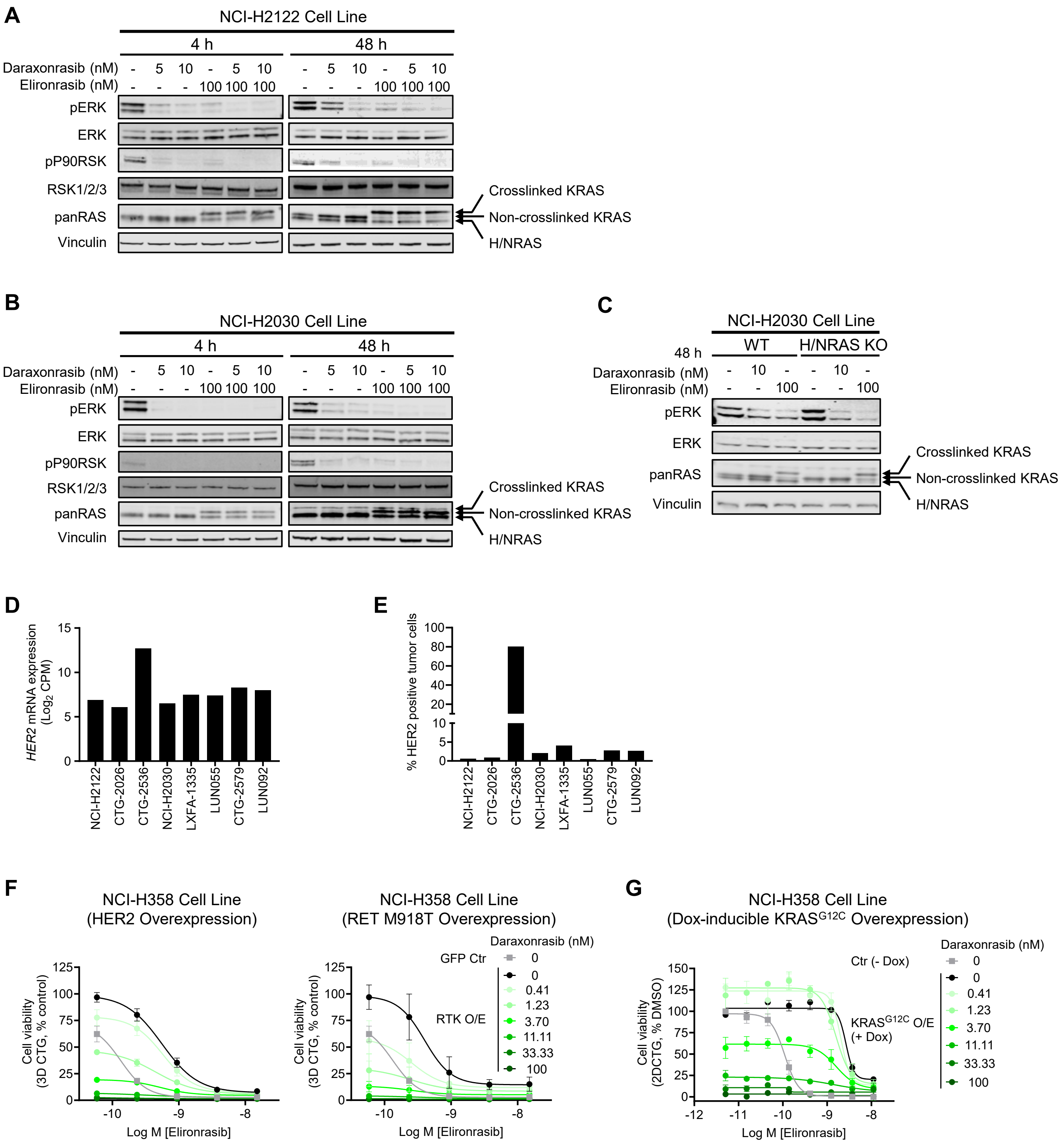

Supplementary Figure 2. The RAS(ON) inhibitor doublet demonstrates combinatorial benefit *in vitro*. **A,** Western blot analyses of KRAS pathway targets in NCI-H2122 (*KRAS*$^{G12C/G12C}$ *STK11*^{mut} *KEAP1*^{mut}, NSCLC) and **B-C,** NCI-H2030 (*KRAS*$^{G12C/G12C}$ *STK11*^{mut} *KEAP1*^{mut}, NSCLC) WT and H/NRAS knockout (KO) cancer cells treated with indicated compounds at specific time points. **D,** mRNA expression levels (Log_2 CPM) of *HER2* from RNA-seq analysis in 8 *KRAS*^{G12C} NSCLC subcutaneous xenograft tumors at baseline included in the tumor response waterfall plot and Kaplan-Meier analysis in Figure 1. **E,** The quantification of HER2 IHC staining in tumor areas of 8 *KRAS*^{G12C} NSCLC subcutaneous xenograft tumors at baseline as shown in Figure 2C. **F,** NCI-H358 (*KRAS*$^{G12C/WT}$, NSCLC) cancer cells expressing doxycycline-inducible exogenous HER2, RET M918T, or GFP control DNA constructs were treated with elironrasib and the indicated concentrations of daraxonrasib for 120 hours, and proliferation was measured by 3D CTG. Data points are the mean of technical duplicates normalized to vehicle control. Error bars indicate s.d. Data shown are representative of two independent experiments. **G,** NCI-H358 (*KRAS*$^{G12C/WT}$, NSCLC) cancer cells expressing exogenous KRAS^{G12C} with the induction of doxycycline (Dox) or control cells without Dox treatment were treated with elironrasib and the indicated concentrations of daraxonrasib for 120 hours, and proliferation was measured by 2D CTG. Data points are the mean of technical duplicates normalized to control. Error bars indicate s.d.

Supplementary Figure 3

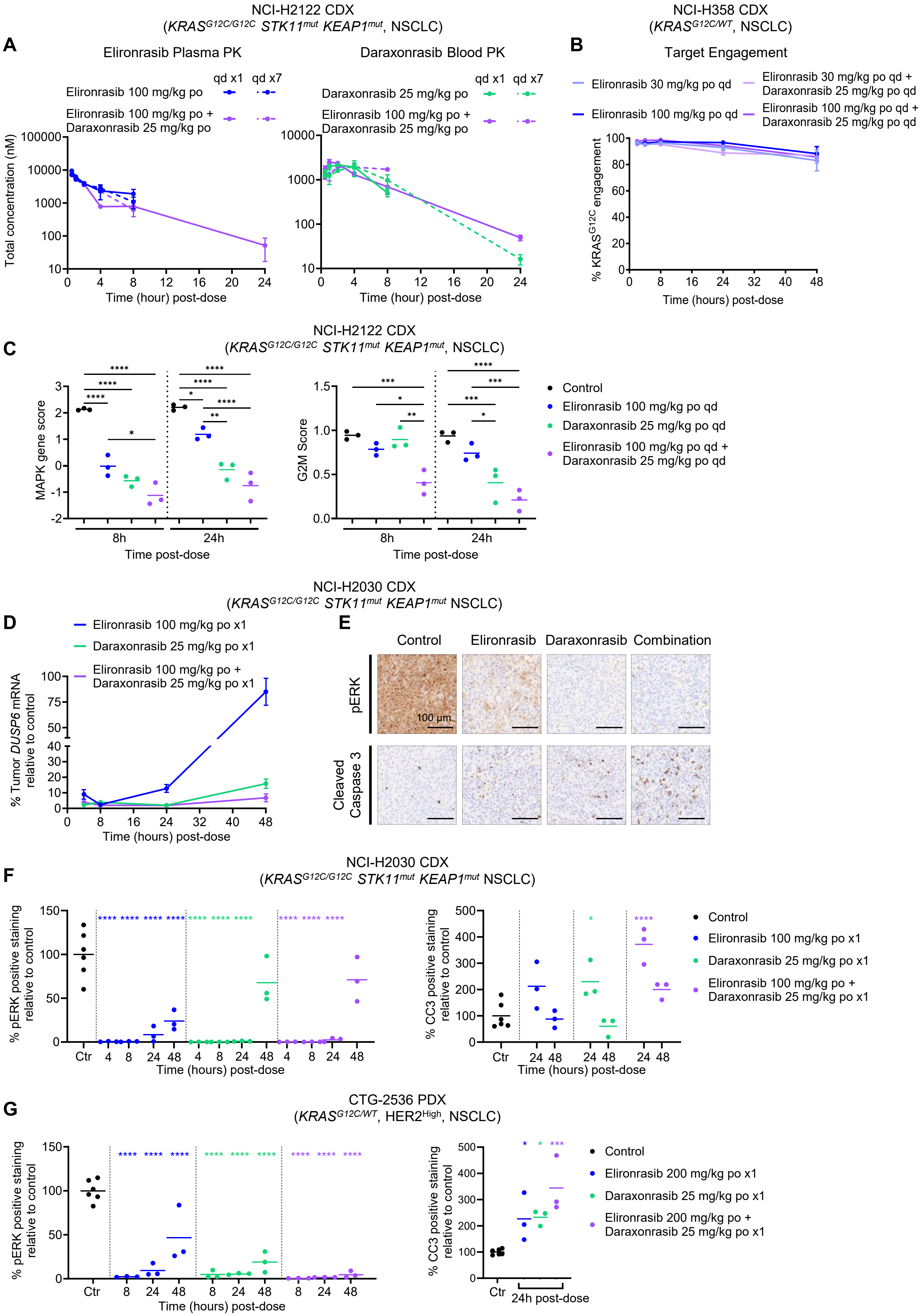

Supplementary Figure 3. The RAS(ON) inhibitor doublet demonstrates combinatorial benefit *in vivo*. **A,** Systemic PK profiles of elironrasib and daraxonrasib in NCI-H2122 ($KRAS^{G12C/G12C}$ $STK11^{mut}$ $KEAP1^{mut}$, NSCLC) subcutaneous xenograft tumor-bearing BALB/c nude mice. Tumor-bearing mice were treated with a single dose (qd x1, solid lines) or 7 consecutive daily doses (qd x7, dashed lines) of elironrasib at 100 mg/kg and daraxonrasib at 25 mg/kg as single agents or in combination. Plasma and blood were harvested at indicated time points (n = 2-3 per time point). PK profiles are shown as elironrasib concentration in plasma and daraxonrasib concentration in blood over time. Data from elironrasib single agent, daraxonrasib single agent and RAS(ON) inhibitor doublet groups are plotted in blue lines, green lines, and purple lines, respectively. Values are plotted as mean ± SEM. **B,** Target engagement of $KRAS^{G12C}$ protein by elironrasib in NCI-H358 ($KRAS^{G12C/WT}$, NSCLC) subcutaneous xenograft tumors, shown as the percentage of crosslinked $KRAS^{G12C}$ by elironrasib relative to controls. Tumor-bearing mice were treated with 7 consecutive daily doses of elironrasib at 30 or 100 mg/kg as single agent (blue lines) or in combination with daraxonrasib at 25 mg/kg (purple lines). Tumors were harvested at indicated time points (n = 3 per time point). Values are plotted as mean ± SEM. **C,** Transcriptomic analysis of MAPK pathway and G2M gene expression signatures based on the bulk RNA sequencing of NCI-H2122 ($KRAS^{G12C/G12C}$ $STK11^{mut}$ $KEAP1^{mut}$, NSCLC) subcutaneous xenograft tumors. Tumor-bearing mice were treated with 7 consecutive daily doses of elironrasib at 100 mg/kg and daraxonrasib at 25 mg/kg as single agents or in combination. Tumors were harvested at indicated time points (n = 3 per time point). **D,** PD in NCI-H2030 ($KRAS^{G12C/G12C}$ $STK11^{mut}$ $KEAP1^{mut}$, NSCLC) subcutaneous xenograft tumors, shown as the relative change in human *DUSP6* mRNA expression. Tumor-bearing mice were treated with a single dose of elironrasib at 100 mg/kg and daraxonrasib at 25 mg/kg as single agents or in combination. Tumors were harvested at indicated time points (n = 3 per time point). Data from elironrasib single agent, daraxonrasib single agent and RAS(ON) inhibitor doublet groups are plotted in blue lines, green lines, and purple lines, respectively. Values are plotted as mean ± SEM. **E-F,** Histopathology analysis of NCI-H2030 subcutaneous xenograft tumors treated with a single dose of elironrasib at 100 mg/kg and daraxonrasib at 25 mg/kg as single agents or in combination and collected at indicated time points (n= 3 per time point). (**E**) Representative images are shown at 40× magnification from samples closest to the mean of the group. Tumors used for representative images of pERK and CC3 were collected at 24 hours post a single dose of RAS(ON) inhibitors. Scale bars, 100 μm. (**F**) pERK and CC3 IHC staining in tumor areas were quantified and compared with vehicle using one-way ANOVA followed by Dunnett multiple comparison test (*, P < 0.05; ****, P < 0.0001). **G,** pERK and CC3 IHC staining in tumor areas of CTG-2536 subcutaneous PDX tumors as shown in Figure 3F were quantified and compared with vehicle using one-way ANOVA followed by Dunnett multiple comparison test (*, P < 0.05; ***, P < 0.001 ; ****, P < 0.0001).

Supplementary Figure 4

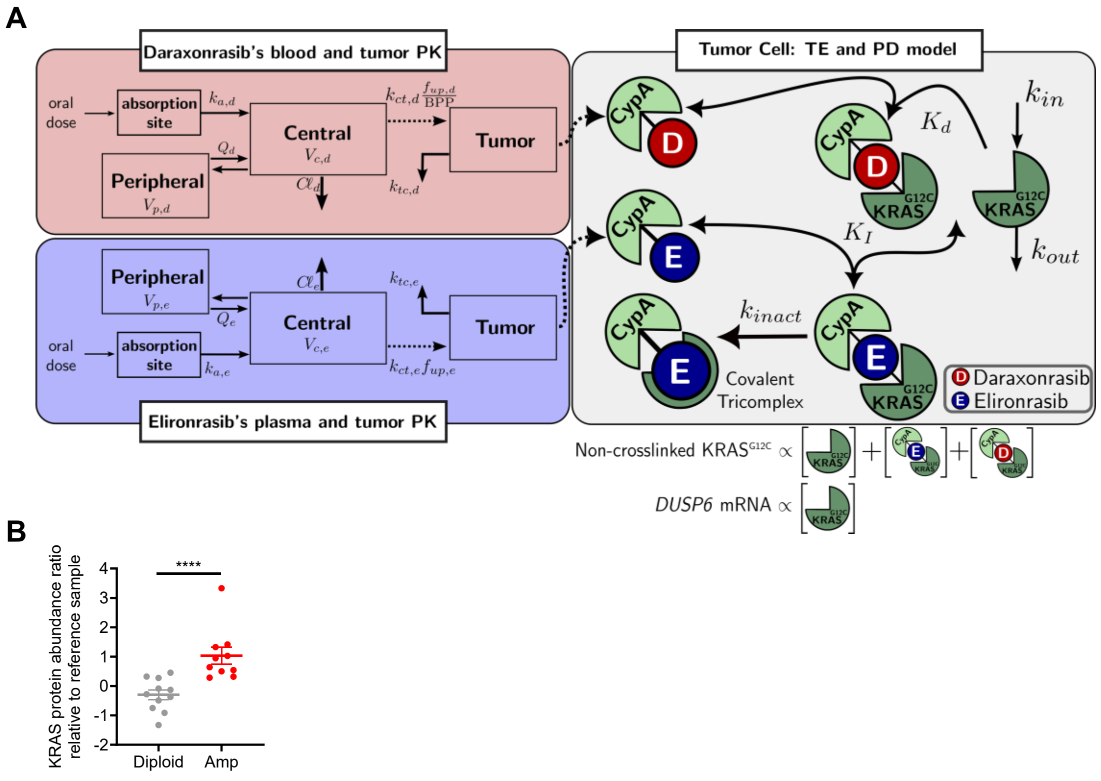

Supplementary Figure 4. Graphical representation of the combined PK/TE/PD model. **A,** The model consists of a cascade of two uncoupled PK models which simultaneously drive a combined TE/PD model. The blood PK of daraxonrasib and plasma PK of elironrasib are described by a two compartmental model, which drives an effect compartment for the tumor PK. A schematic model of a tumor cell illustrates the turnover of $KRAS^{G12C}$, its binding by either agents, and the crosslinking by elironrasib. The TE data corresponds to the sum of all the non-crosslinked $KRAS^{G12C}$ species, while *DUSP6* mRNA data correspond to the free $KRAS^{G12C}$ species. **B,** KRAS protein expression in *KRAS* non-amplified (Diploid, n=11) and amplified (Amp, n=10) *KRAS*-mutant NSCLC cell lines from CCLE cohort accessed through cBioportal. Protein abundance ratios relative to the reference sample are obtained from cBioportal and plotted as mean ± SEM. Nonparametric Mann-Whitney test was used to compare groups (****, $P<0.0001$).

Supplementary Figure 5

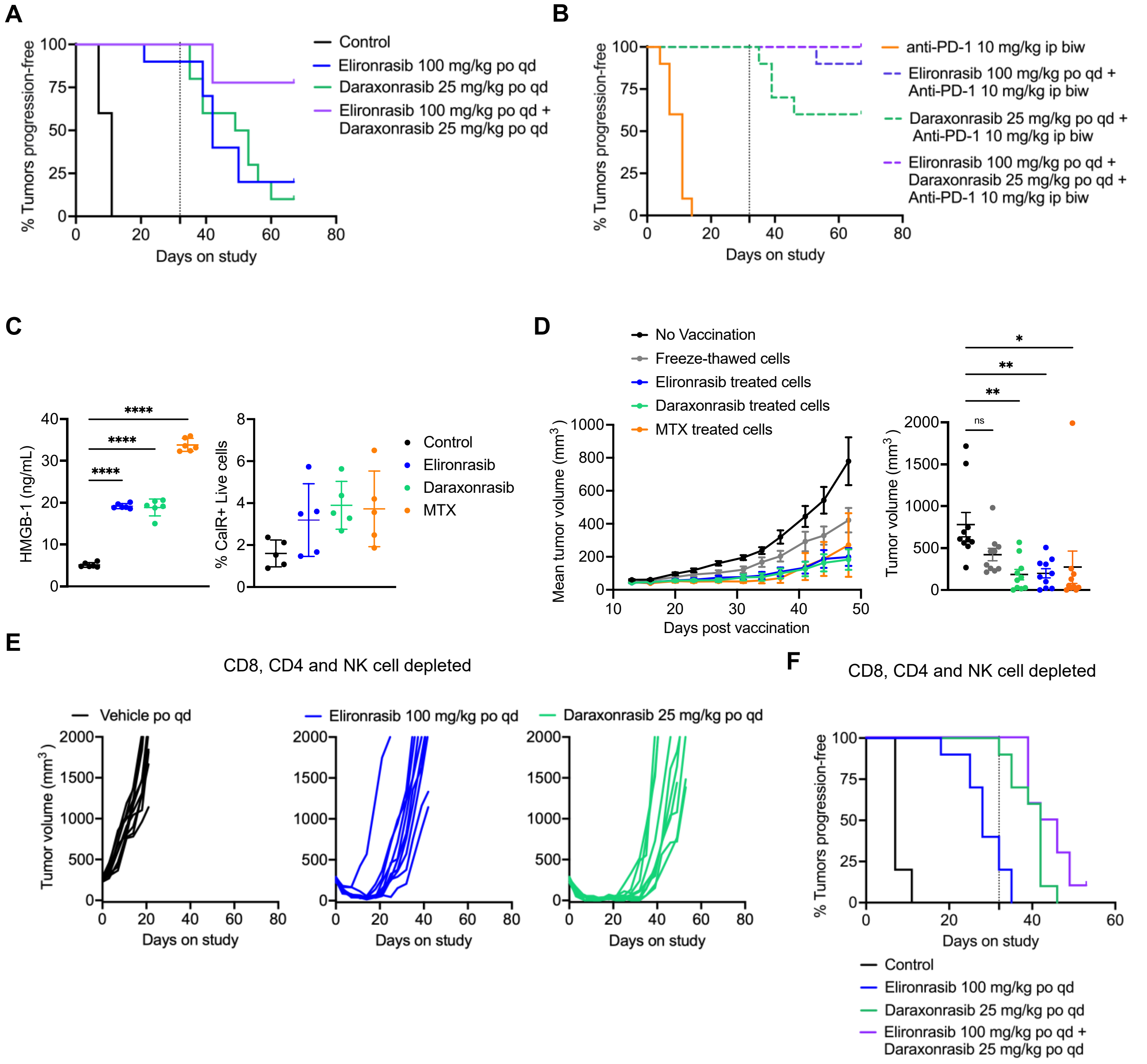

Supplementary Figure 5. Elironrasib and daraxonrasib synergize and drive ICD and immune dependent CR in the KPAR1.3 model. **A-B** Kaplan–Meier analyses of time to tumor doubling upon treatment for 32 days with **A** Vehicle, elironrasib at 100 mg/kg, daraxonrasib at 25 mg/kg and the RAS(ON) doublet. **B,** Anti-PD-1 as single agent at 10 mg/kg ip biw or in combination with elironrasib, daraxonrasib and the RAS(ON) doublet. **C,** HMGB-1 release, measured using the Lumit HMGB-1 Immunoassay in KPAR cells treated for 48 hours with indicated compounds *in vitro* and Calreticulin externalization measured by flow cytometry at 24h after treatment. % of CalR+ live cells was quantified. Cells were treated *in vitro* with the Cmax doses of daraxonrasib and elironrasib, as determined in preclinical studies in mice when dosed at 25 mg/kg and 30 mg/kg, respectively (9 nM and 43 nM respectively), and 1 µM of mitoxantrone (MTX). Elironrasib and daraxonrasib increased (to a similar extent) HMGB-1 release (4-fold compared to control) and CalR externalization (2-fold compared to control), and the activity was comparable to the known ICD inducer mitoxantrone (MTX, 7-fold and 2-fold, respectively) **D,** Tumor volume mean (left) and tumor volumes at day 48, the last day with all mice on study (right) upon vaccination with KPAR1.3 G12C cells pre-treated with indicated regimens in vitro (72 h treatment with 1 µM elironrasib or daraxonrasib or 48h with 5 µM MTX) rechallenged with KPAR1.3 G12C cells on the contra-lateral side. **E** Tumor growth and **F**, Kaplan–Meier analyses of time to tumor doubling upon treatment for 32 days with elironrasib at 100 mg/kg or daraxonrasib at 25 mg/kg po qd in the presence of CD4, CD8 T cell and NK cell depleting antibodies administered at 10 mg/kg at day -1, 0 and every 6 days post dosing start. Asterix indicates analysis was performed using one-way ANOVA. For all statistical analysis *$p < 0.05$, **$p < 0.01$, ***$p<0.001$.

Supplementary Figure 6

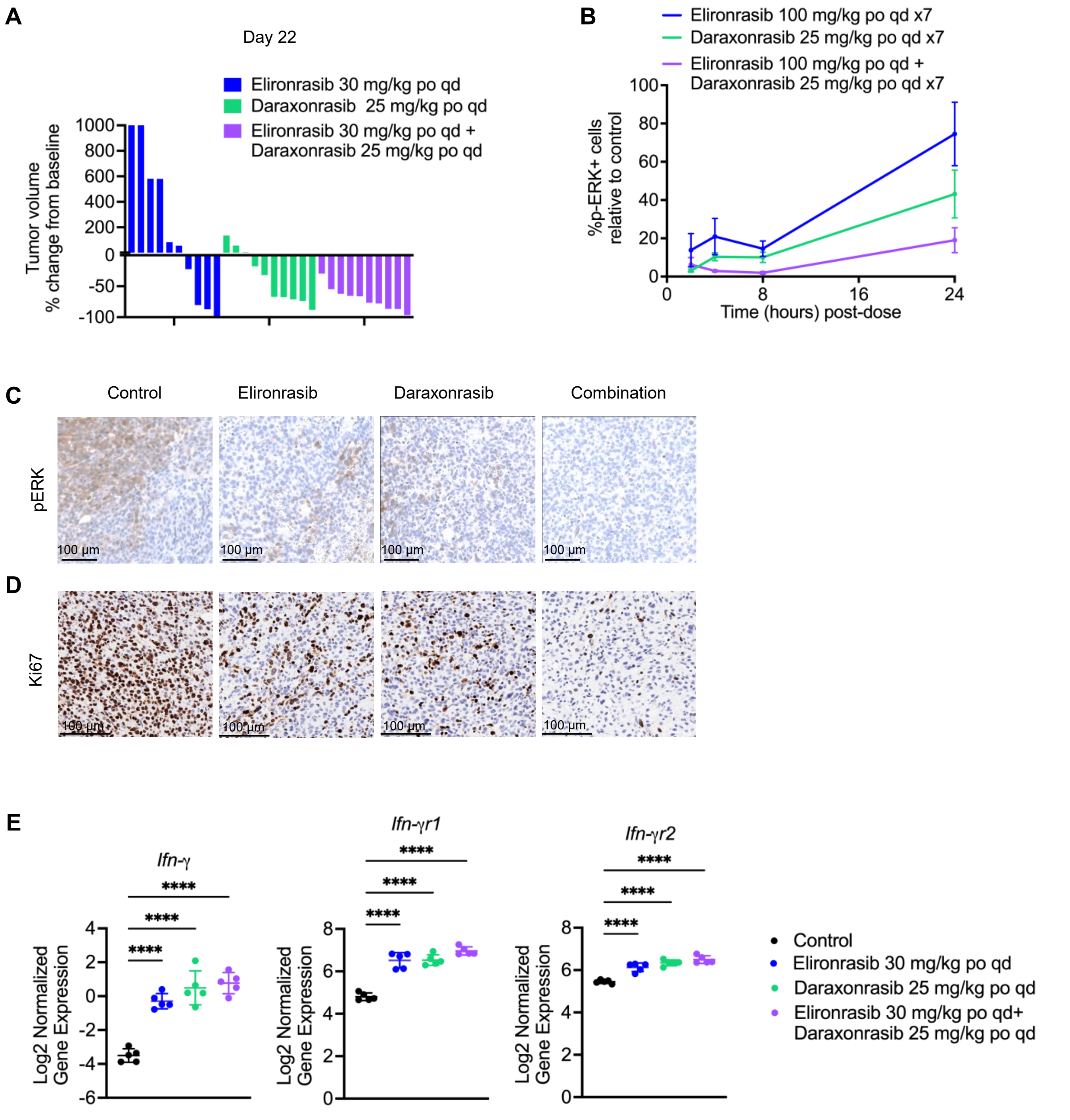

Supplementary Figure 6. The RAS(ON) doublet increases depth of response in the 3LL-ΔNRAS model. **A** Tumor response waterfall plots upon treatment with elironrasib at 30 mg/kg or daraxonrasib at 25 mg/kg po qd for 22 days (last time point all mice were on study). **B**, PD of elironrasib, daraxonrasib and the RAS(ON) doublet in subcutaneous tumors shown as the relative change in pERK expression in tumor cells. Tumor-bearing mice were treated for 7 days with elironrasib at 30 mg/kg, daraxonrasib at 25 mg/kg and the RAS(ON) doublet po qd and tumors were collected at indicated time points. **C**, Representative IHC image (scale bar 100 um) of pERK in tumors at 24 hours post single dose of elironrasib at 30 mg/kg, daraxonrasib at 25 mg/kg and the RAS(ON) doublet. **D**, Representative IHC image (scale bar 100 um) of Ki67 at 24 hours post 8 days repeat dosing with elironrasib at 30 mg/kg, daraxonrasib at 25 mg/kg and the RAS(ON) doublet. **E**, Log2 normalized expression of indicated genes based on bulk RNA sequencing of whole tumors collected at 24 hours post 8 days of treatment.

Supplementary Figure 7

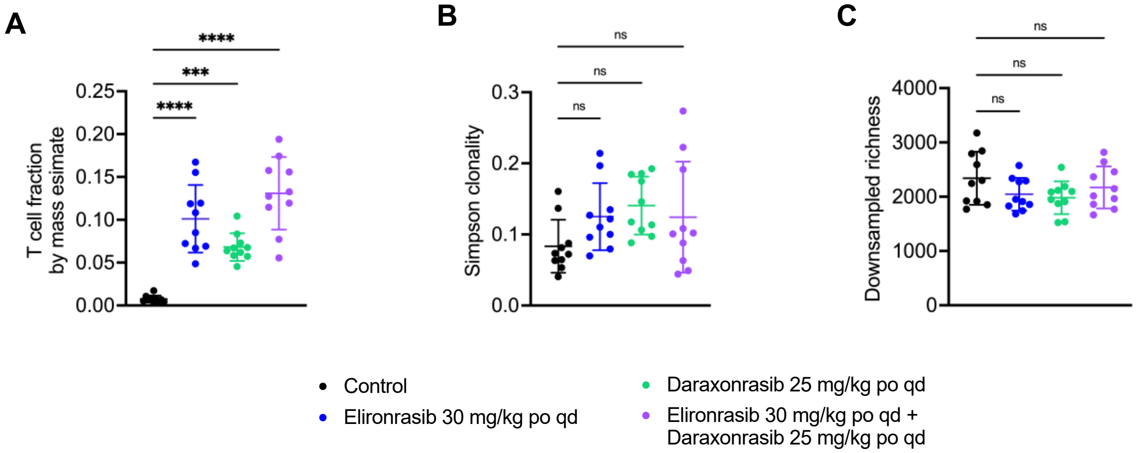

Supplementary Figure 7. Immuno-sequencing (TCRB assay) of gDNA extracted from 3LL-ΔNRAS tumors collected at 24 hours post 8 days of daily oral treatment with vehicle, elironrasib at 30 mg/kg or daraxonrasib at 25 mg/kg or the RAS(ON) doublet. **A,** T cell abundance calculated as total T cells divided by the total nucleated cells estimated from the sample mass. **B,** Evenness of clone frequencies in the repertoire represented as simpson clonality. **C,** Number of unique clones (rearrangements) represented as richness down sampled to a common number of templates. Analysis was performed using one-way ANOVA. For all statistical analysis *p < 0.05, **p < 0.01, ***p<0.001, ****p<0.0001**.**
